# Neuronal oscillations form parietal/frontal networks during contour integration

**DOI:** 10.3389/fnint.2014.00064

**Published:** 2014-08-13

**Authors:** Marta Castellano, Michael Plöchl, Raul Vicente, Gordon Pipa

**Affiliations:** ^1^Department of Neuroinformatics, Institute of Cognitive Sciences, University of OsnabrückOsnabrück, Germany; ^2^Faculty of Mathematics and Computer Science, Institute of Computer Science, University of TartuTartu, Estonia

**Keywords:** oscillations, parietal cortex, feature binding, contour integration, visual perception

## Abstract

The ability to integrate visual features into a global coherent percept that can be further categorized and manipulated are fundamental abilities of the neural system. While the processing of visual information involves activation of early visual cortices, the recruitment of parietal and frontal cortices has been shown to be crucial for perceptual processes. Yet is it not clear how both cortical and long-range oscillatory activity leads to the integration of visual features into a coherent percept. Here, we will investigate perceptual grouping through the analysis of a contour categorization task, where the local elements that form contour must be linked into a coherent structure, which is then further processed and manipulated to perform the categorization task. The contour formation in our visual stimulus is a dynamic process where, for the first time, visual perception of contours is disentangled from the onset of visual stimulation or from motor preparation, cognitive processes that until now have been behaviorally attached to perceptual processes. Our main finding is that, while local and long-range synchronization at several frequencies seem to be an ongoing phenomena, categorization of a contour could only be predicted through local oscillatory activity within parietal/frontal sources, which in turn, would synchronize at gamma (>30 Hz) frequency. Simultaneously, fronto-parietal beta (13–30 Hz) phase locking forms a network spanning across neural sources that are not category specific. Both long range networks, i.e., the gamma network that is category specific, and the beta network that is not category specific, are functionally distinct but spatially overlapping. Altogether, we show that a critical mechanism underlying contour categorization involves oscillatory activity within parietal/frontal cortices, as well as its synchronization across distal cortical sites.

## Introduction

A fundamental ability of the neural system is to integrate visual features into coherent percepts, whereby the segments belonging to an object boundary are perceptually grouped (Wertheimer, 1923; Field et al., 1993). One particular instance of perceptual grouping, where a coherent percept arises through the integration of a single stimulus feature, is contour integration, where a set of local elements are integrated to a common contour due to its relative orientation (Field et al., 1993). Evidence from psychophysical (Field et al., 1993; Li and Gilbert, 2002; Mathes et al., 2006), physiological (Li et al., 2006), and neuroimaging studies (Altmann et al., 2003; Kourtzi et al., 2003) report enhanced activity within early visual cortex, suggesting that contour integration can be mediated within the primary visual cortex itself, giving form to the saliency hypothesis. A complementary set of studies that argue for this hypothesis is that contour detection performance strongly depends on the spatial organization of the local elements (reviewed in Hess and Field, 1999), up to the extent that behavioral performance is thought to be explained by the anatomy of the visual cortex (Field et al., 1993).

Simultaneously, neuroimaging, lesion, and electrophysiology studies provide evidence that the processing and perception of visual stimulus entails the participation of multiple and widespread brain areas, emphasizing the selective role of early visual cortices (Hubel and Wiesel, 1962; Mishkin et al., 1983), parietal cortex (Tallon-Baudry et al., 1997; Volberg and Greenlee, 2014), and frontal cortices (Foxe and Simpson, 2002; Morgan et al., 2013) on this process. In particular to perceptual grouping, several studies suggest the involvement of higher-order areas the integration process as contour detection and its neural signatures arising in early visual cortex seem to be modulated by the task requirements, including attentional demands (Roelfsema et al., 2004), perceptual learning (Li et al., 2006), or perceptual noise within the contour (Mathes et al., 2006). Altogether, these studies suggest that perceptual grouping, as well as contour integration, seem to involve the processing within non-primary visual cortex.

Of particular interest for the understanding of the neural mechanisms that mediate perceptual grouping is neural oscillatory activity. Local enhancement of oscillatory activity within early visual cortices has been associated with visual processing and perceptual grouping itself through intracranial recordings (Gray et al., 1989; Fries, 2009; Uhlhaas et al., 2009), and human EEG/MEG studies (Lutzenberger et al., 1995; Tallon-Baudry et al., 1997; Hoogenboom et al., 2006; Donner et al., 2007; Volberg et al., 2013). But it is not only synchronization in local cortical areas that is relevant for perceptual grouping. Recent studies report transient synchronization between parietal and frontal cortices: low frequency oscillations (7–14 Hz) have been proposed to coordinate activity between disperse cortical areas during visual processing (Tallon-Baudry et al., 2001; Sehatpour et al., 2008), while enhanced synchronization within the gamma frequency band (>30 Hz) has been associated with visual integration of segregated features and cross-modal integration across independent processing streams (Palva et al., 2005; Hipp et al., 2011). To this end, neural synchronization has been proposed as the mechanism by which visual information is integrated into a percept, grouping visual features at both localized cortical areas and across distributed cortical areas (Singer, 1999; Varela et al., 2001).

**Figure 1:**
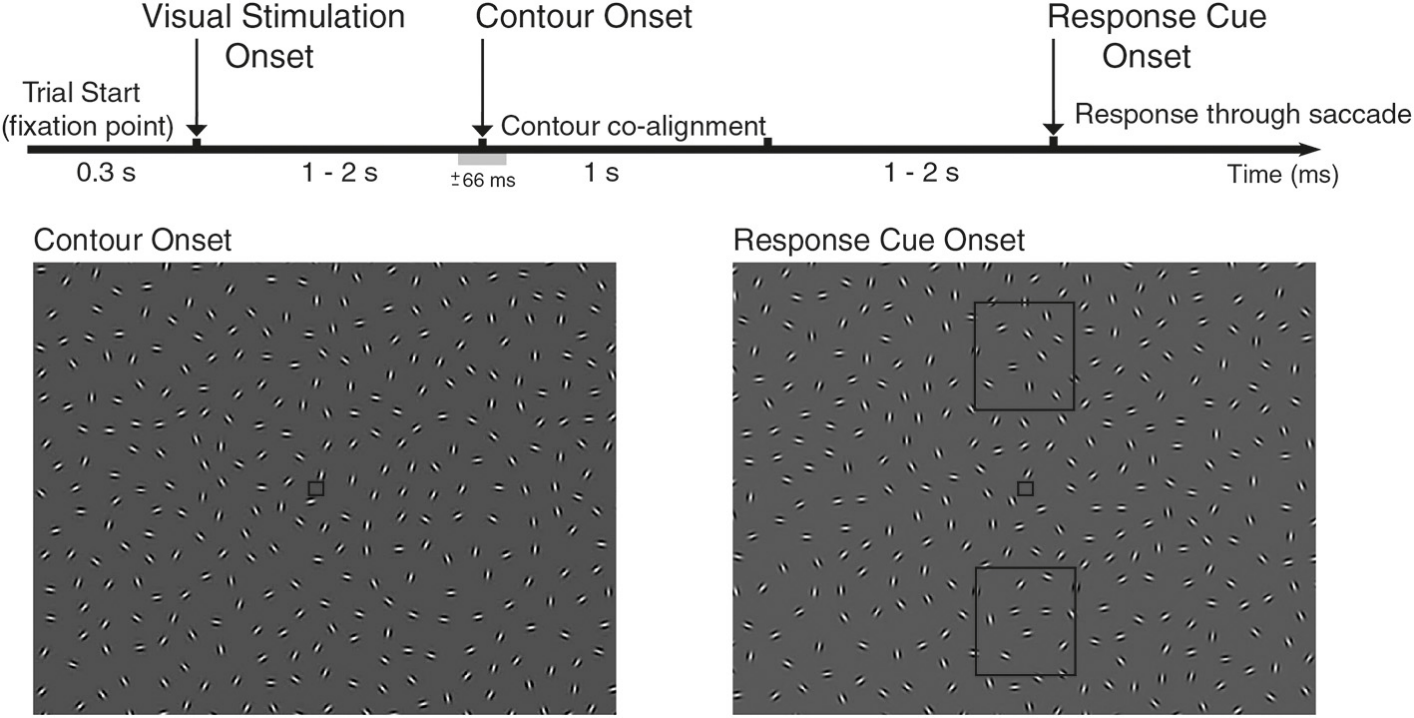
**Experimental design of the study**. EEG was recorded while subjects performed a contour integration task, where participants were instructed to identify two different orientations of an oval contour. The contour would appear on half the trials (contour vs. non-contour trials), randomly on the left/right hemifield in varying locations in respect to horizontal view, lasting for 1 s. Each trial starts with the appearance of a fixation point, followed with a field of Gabor elements (Visual Stimulation Onset, VS) that were continuously changing its orientation (see Video 1 for contour trials and 2 for non-contour trails). After a delay period, a subset of Gabor elements co-align forming an oval-like contour that pointed up/down (Contour Onset, CO; ±66 ms range of contour visibility). Note that in this diagram, the contour is shadowed to increase visibility. Subjects reported identification of the contour via saccade after the response cue onset, indicated with the appearance of two rectangles (up/down) that mark the target location of the saccade (Response Cue Onset, RC). Subjects were instructed to maintain fixation until the appearance of the report cue onset and forced to perform saccade in non-contour trials. Throughout the study, we analyzed neural activity associated with the different behavioral events: VS-CO-RC and with two behavioral conditions associated with CO event (contour and non-contour trials).

Despite the widespread advances to understand the mechanisms by which the neural system performs perceptual grouping tasks, it is not yet clear how both local and long-range oscillatory activity leads to the integration of visual features into a coherent percept (Engel et al., 2001). Here, we investigate whether oscillatory activity within visual cortex predicts perceptual grouping of a visual stimuli, and whether and how perceptual grouping modulates synchronized activity across distributed neuronal populations. To test this, we recorded EEG from human subjects performing a contour categorization task, where co-aligned local elements (i.e., Gabor elements) must be linked and further classified (see Figure 1, Field et al., 1993). Our assumption is that with the use of a contour categorization task, perceptual grouping is be described as a two-stage process, where the local elements that form contour must be linked into a coherent structure, which is then further processed and manipulated to perform the categorization task. Our assumption can be framed in both the incremental grouping theory (Roelfsema, 2006) and perceptual matching theories (Herrmann et al., 2004; Watt et al., 2008), where perceptual grouping is described as a two stage process where the linking of the local elements is followed by a further processing that require high-order cortical areas (see also Mack and Palmeri, 2010; Kourtzi and Connor, 2011). As such, the usage of a categorization task as a framework for the study of perceptual grouping allows for a disentanglement of the linking process of local elements (Contour Linking Process) and the further contour processing for its categorization (Contour Processing), which may involve several secondary processes, such as top-down attentional selection (Mesulam, 1999; Buschman and Miller, 2007; Siegel et al., 2008; Van Ede et al., 2012), memory matching (Herrmann et al., 2004), or the targeting of the contour (VanRullen and Thorpe, 2001). In particular, we expect the involvement of fronto-parietal areas that are proposed to mediate the formation and selection of behaviorally relevant stimulus (Sato and Schall, 2003). Furthermore, in our task, contour formation is a dynamic process where contour is continuously morphing (see Video 1 for a contour trial and 2 for a non-contour trial), so that the appearance of the contour is, for the first time, not associated with a sudden onset of the visual stimuli. This paradigm, in combination with a new analysis approach, allows for the identification of cortical areas that are associated with different behavioral events, such as the processing of visual stimulus, the linking of contour elements, the further contour processing for correct categorization and saccade planning. In the following, we describe the oscillatory mechanisms involved in these processes and their modulations within and across spatially distinct cortical networks.

## Materials and methods

### Participants and stimuli

A total of 15 participants (9 female and 6 male, aged 18–32 years) gave informed written consent to participate in the study, approved by the local ethics committee and conducted in accordance with the Declaration of Helsinki and national guidelines. Participants reported normal or corrected-to-normal vision, with no history of neurological or psychiatric illness. At each trial, participants were presented with a frame of 335 randomly oriented Gabor elements (each spanning 0.5° of visual angle) whose orientation was continuously changing [2° ± 2.6° (mean ± std) per frame], leading to the perception of smoothly rotating Gabor elements. During the trial, Gabor elements would keep rotating and, on half of the trials, the orientation of 22 Gabor elements would co-align to form an oval-like contour that spans 11.3° of the visual field (see Video 1 for contour trials and Video 2 for non-contour trials,.avi and.mpeg format). The modulation of the angle of Gabor elements introduces temporal evolution on the contour formation, so that collinear contours are continuously morphing and perceived as dynamic stimuli with smooth progression between stimulation frames. The contour is an oval-like shape, so that the curvature at both asymmetric ends was rather similar (see Figure 1). Contours appeared in 50% of the trials, on either the left or right hemifield (25% of total trials) on five different positions relative to the horizon (see Figure S1). Stimuli were displayed on a DELL UltraSharp LCD monitor, VGA mode, 1024 × 768 pixel resolution, frame rate 60 Hz. The participants viewed the screen binocularly at 60 cm distance in a room with dim light and constant luminance.

### Behavioral task

The task of the participants was to identify two different spatial orientations of the same oval-like contour, and report its orientation in a two-alternative forced choice task (2AFC, up/down response, Mathes et al., 2006). Contours appear 50% of the trials, and the narrow side of the oval corresponds to the pointing direction of the contour (oriented either up or down the screen). The identification of the contour's direction was reported through a voluntary saccade toward the pointing direction of the oval after the response cue (see Figure 1), and subjects were required to make a random choice of an up or down eye movement in the case where no contour was identified, which include non-contour and false negative trials due to high noise. This last constraint forces the participant to plan and execute an eye movement in every trial, so that trials where contour orientation is reported cannot be distinguished from the other trials based on the resulting eye movement. Each trial starts with the appearance of a fixation dot (spanning 0.3° of visual angle, see Figure 1). After a 300 ms, visual stimulation starts with a full-frame field of randomly oriented Gabor elements, and lasts either 1.03, 1.50, or 1.97 s. This is the first behavioral event, namely, the visual stimulation onset or VS event. After this period, a subset of 22 Gabor elements co-align to form the described oval contour, and stay in co-alignment for 1.03 s. Note that while the orientation of Gabor elements changes at the same rate (2° ± 2.6° (mean ± std) per frame), they remain aligned to the underlying contour path, see Video 1 for an example of a contour trial. This is the second behavioral event considered for analysis: the contour onset (CO) event. As contours appear on 50% of the trials, the CO event has two behavioral conditions associated: contour vs. non-contour trials. After a random period (either 1.03, 1.50, or 1.97 s), the response cue appears (two rectangular shapes up/down the fixation point, see Figure 1), marking the moment when participants are required to report the categorization of the contour by performing a saccade toward the response rectangles, located either up/down of the fixation point (RC event). Each participant was required to respond to a total of exactly 720 valid trials, during which EEG and eye movements were recorded. Each trial starts with the appearance of a fixation point, where participants were instructed to fixate on during the total length of the trial. Loss of fixation lead to a premature end of the trial, where loss of fixation is defined as saccades with an amplitude larger than 1.25°. Analyses were performed in Matlab (MathWorks, Natick, MA) with custom code and several open-source toolboxes: field trip (Oostenveld et al., 2011) and EEGLab (Delorme and Makeig, 2004).

### Contour visibility as a function of Gabor orientation noise

Given that the contour within contour trials arises through continuously morphing Gabor elements, the time at which the contour is identified at each trial may vary. We account for this variability around the CO event in two ways. First, we define that the CO time *t* = 0 marks the time point at which the contour can be identified with a probability of 0.7, on average over subjects. Second, we define a range of contour visibility, which delineates the time interval within the trial at which the contour will be identified with a probability above chance. For that, we estimated a psychometric curve that defined the subject's performance in the contour categorization task for the different degrees of co-alignment of the Gabor elements. This experiment was conducted with a different set of 7 male participants, aged 24–29 who gave informed consent to participate in the study, approved by the local ethics committee and conducted in accordance with the Declaration of Helsinki and national guidelines (see Supplementary Materials for details). In short, the psychometric function associates the degree of co-alignment Φ with a identification probability so that, to obtain the probability of a contour being identified at time *t*, with a known degree of co-alignment Φ, we inverse the psychometric function: on average for all participants, with an alignment of Φ = 50°, the contours are identified by chance, which corresponds to 66 ms before the CO event.

### Data recording, preprocessing and neural source reconstruction

Synchronization of stimulus presentation, eye-tracking, and EEG recordings was controlled via ViSaGe software (Cambridge Research Systems Ltd.). Eye movements were recorded monocularly by EyeLink 1000 (SR Research Ltd.). Electroencephalogram was recorded via 64 channel ActiCap (Brain Products GmbH). Electrode impedances were kept below 5 kΩ. We averaged referenced the raw EEG data Next, the data were high-pass filtered at 1 Hz by a FIR filter to remove very low frequency and constant trends. Epoching was performed from −1 to 2 s around the behavioral event of interest (i.e., VS, CO, and RC). After epoching, we corrected for baseline drifts by subtracting the mean baseline [(−0.5, 0) s before behavioral event of interest]. Trials with strong muscle activity were identified and removed by visual inspection. While this approach removed severe artifacts, we decided to reduce possible remaining artifacts by rejecting trials with extreme values, linear trends, improbable data and highly negative kurtosis (as suggested in Delorme et al., 2007). To further control for the presence of microsaccade artifacts on our data, we performed microsaccade detection on preprocessed and epoched data by an algorithm published by Engbert and Mergenthaler (2006). Trials with microsaccades were discarded of further analysis.

Preprocessing resulted in 330 ± 20 (mean ± std) contour trials and 210 ± 14 non-contour trials per subject. Sources of neural activity were localized from the Independent Component Analysis (ICA) estimates, computed on preprocessed EEG data an obtaining a total of 64 signal sources per subject (maximum number of IC given the number of EEG electrodes, see Supplementary Materials). Dipole localization of the resulting components topography is performed using DIPFIT2.2 plugin on EEGLab (Delorme and Makeig, 2004), based on a three-shell boundary element model (BEM) on a MNI standard brain template. Source localization on ICs instead of EEG signals reduces several of the factors that add errors on the dipole localization (e.g., environmental noise, non-linear interference between sources, etc.), increasing accuracy on the source localization (Tarkiainen et al., 2003). Note that source localization error of ICA decomposed signals typically extend from 4.1 to 20 mm (Acar and Makeig, 2013), so that the quantitative localization of the dipoles only provides an approximation for the exact location of the underlying source. Non-neural sources (e.g., localized at the scalp) were discarded of further study, leading to an average of 55 ± 5 neural sources per subject.

### Spectral decomposition and phase locking value

Two spectral decompositions of the ICs were performed. The spectral analysis presented in the spectrograms for high frequency bands (>30 Hz) was computed by a multitaper method, which provides a way to control bias and variance of the spectral estimation by using multiple Slepian tapers (Percival and Walden, 1993). The time-frequency decomposition was computed on epoched data, with a Slepian tapers, in steps of 2 Hz, with a window of 12 cycles per frequency and a spectral smoothing of 1/5 the frequency. The spectral analysis for frequency bands between 5 and 30 Hz was computed using Morlet wavelets, with a width of four cycles per frequency, and steps of 2 Hz. The spectrograms were computed on 90 trials per condition (contour/non-contour) and subject, selected randomly from the available trials. Finally, spectrograms were presented as a power change in respect to the baseline (−0.5 to 0 s relative to event of interest), and then averaged over trials and subjects.

Spectral analysis for the detection of task-relevant neural sources was performed through a discrete multi-scale wavelet transform using Daubechies wavelets of order 4 (DWT). The instantaneous frequency was computed on epoched data, leading to 5 frequency intervals (5.6, 11.16, 22.32, 44.6, and 89.3 Hz mean frequency). The instantaneous phase was extracted by the Hilbert transform. The phase locking value (PLV) (Lachaux et al., 2000), was computed on the instantaneous phase obtained from the DWT spectral decomposition for two different behavioral conditions (contour and non-contour trials). Phase synchronization was estimated between each pairwise combination of neural sources and frequency bands on 90 trials per behavioral condition (contour/non-contour trials, selected randomly from the available trials), with a resolution of 1 ms.

### Detection of task-related neural sources

**Figure 2:**
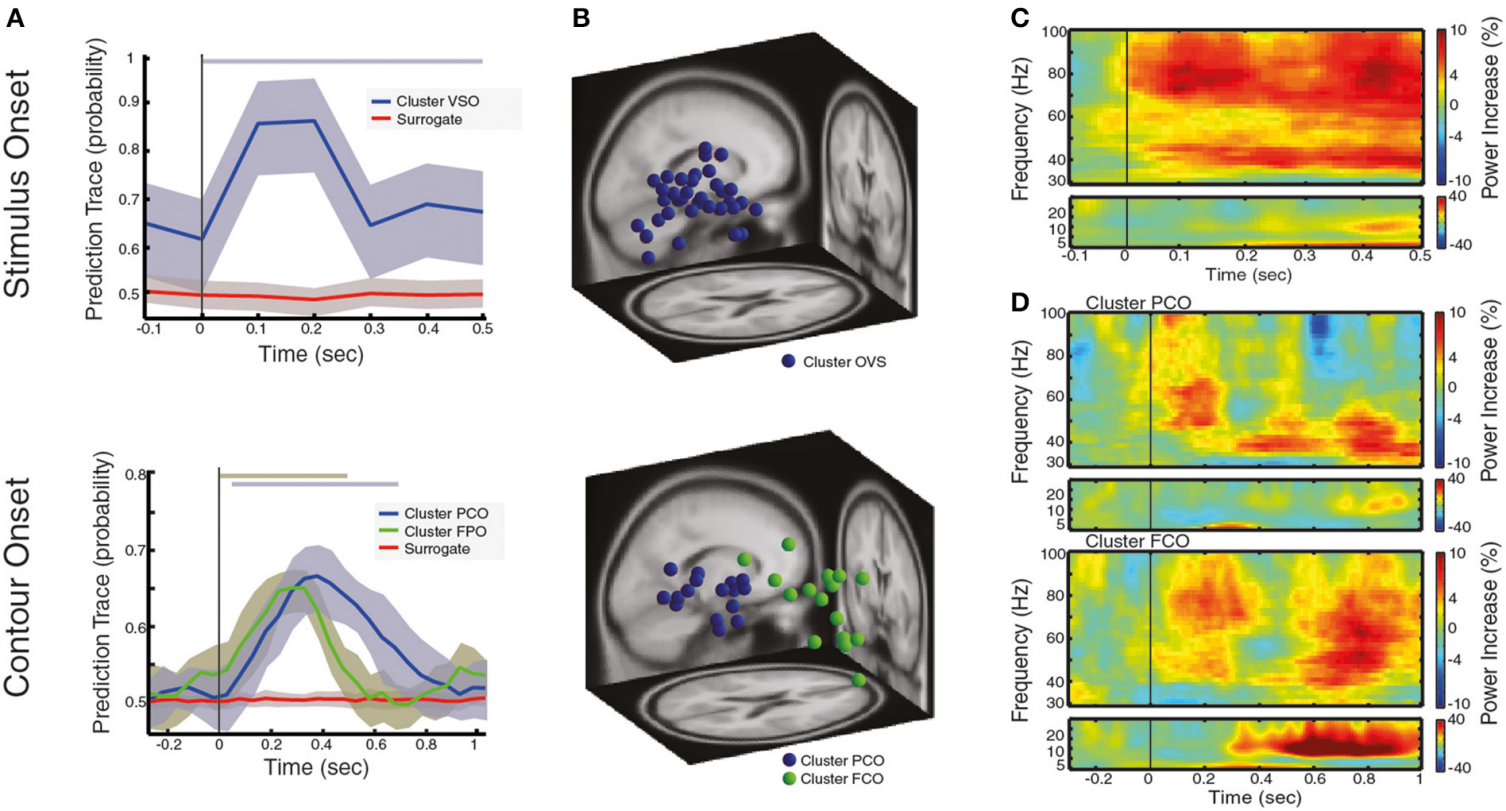
**Local oscillatory activity during the processing of visual stimulus and contour categorization**. **(A)** Prediction traces for neural sources that are associated with the processing of visual stimulus after VS onset (left) and for neural sources that associate with correct contour categorization after CO onset (right). The prediction trace of a neural source is the time-resolved probability of a neural source to belong to a behavioral condition of interest (see text for details). Prediction traces of neural sources across subjects are shown in blue/green (mean ± std), while the prediction traces of surrogate data is shown in red (mean ± std). Significance of prediction traces is shown as a shadow in upper part of the plot, colorcoded for different clusters (*p* < 0.01, corrected for multiple comparison). **(B)** Dipole localization of neural sources associated with the processing of visual stimulus (left) and contour categorization (right), across subjects. In **(C,D)** the spectrogram of contour trials reflecting the power increase in respect to baseline, averaged over neural sources and subjects for **(C)** of neural sources associated with visual stimulation at VS onset and **(D)** neural sources associated with contour categorization cluster PCO (upper) and cluster FCO (lower) at CO onset. Note the range of contour visibility spans ± 66 ms around CO onset (the time interval at which subjects identify the contour better than chance).

The goal of this method is to select neural sources that are associated with our stimulus of interest without any prior assumptions on the data (naive Bayes classifier). Generally, our goal is to compute the likelihood *p(D|c_i_*), namely, the probability that a set of data *D* = {*x^t^*_1_, *x^t^*_2_, …, *x^t^_n_*} belongs to a category *C* = {*c*_1_, *c*_2_} where *n* is the trial number, *t* the time point at which the likelihood is computed, and *C* is a binary behavioral condition of interest. For example, if the goal is to find neural sources that associate with the appearance of stimulus, the classification is a binary classification problem *C* = {0, 1}, where 1 indicates the presence of stimulus. Likewise, other behavioral conditions of interest can be reduced to binary classification problems, such as lateralization of visual stimulation (*C* = {′*left*′, ′*right*′}), or with the direction of eye movement (*C* = {′*up*', ′*down*'}). The likelihood for binary categories was computed via logistic regression (Fahrmeir and Tutz, 2001; Bishop, 2007). In short, logistic regression estimates the relationship between predictors variables and the categorical outcomes *c_i_*, mentioned before. The predictor variables of our logistic regression will be the discrete wavelet transform of the neural sources data, so that *D* = {*x^t^*_1_, *x^t^*_2_, …, *x^t^*_n_} is transformed on the frequency domain such that $\tilde{D}=\{{\tilde{x}}_{1}^{t},{\tilde{x}}_{2}^{t},\ldots ,{\tilde{x}}_{n}^{t}\}$. In other words, the logistic regression problem aims to find a set of parameters $\tilde{w}$ that will establish the following relation: *C* = *w*$\tilde{D}$, where *C* are the categorical outcomes for a time *t* and $\tilde{D}$ is the frequency decomposition of a neural source at time *t*. To further reduce the dimensionality of our model, we included a L1 or Lasso regularization term on the linear regression (Bishop, 2007). In short, within logistic regression, L1 regularization introduces a penalty term α|*w*| on the optimization problem, and that results on forcing some of the parameters to have a zero weight. The penalty term is weighted by the hyper-parameter α. To choose the hyper-parameter α, we trained a set of models with α logarithmically spaced between [0.01, 0.3]. We choose the hyper-parameter α that lead to the model with highest likelihood (Friedman et al., 2010). Fitting the data for logistic regression and Lasso regularization is performed by glmnet (Friedman et al., 2007). Evaluation of the classification performance is computed through the Maximum A Posteriori estimate, so that *C_MAP_* = argmax_*C*_*p*($\tilde{D}$|*c_i_*) (Bishop, 2007). Concatenating the MAP at every time step *t* is what we call the prediction trace (see Figure 2A for an example). The model classification accuracy is validated through repeated random sub-sampling validation, for 100 iterations and a split of the data *D* into 70% training trials and 30% validation trials, so that for each neural source we estimate 100 prediction traces, which are then averaged as to reduce the bias of the model (Kohavi, 1995). By randomizing categorization labels (random labels were generated through random permutation of the trial number), we obtain the likelihood function of the null-hypothesis, which is, the probability distribution that a dataset $\tilde{D}$ belongs to a random class *c_i_* (surrogate data, see Figure 2A for an example). To this end, repeated random sub-sampling validation, for 100 iterations and a split of the data *D* into 70% training trials and 30% validation trials, so that for each surrogate source we estimate 100 prediction traces, from which we obtain a probability distribution of the null hypothesis. The probability distribution of the null-hypothesis was used to compute significance level of the categorization performance, so that if *p*($\tilde{D}$|*c_i_*) is outside the 99% confidence interval of the distribution of the null-hypothesis, the categorization performance is considered significant (*p* < 0.01; Bakeman and Robinson, 2005).

### Long-range synchronization networks

Synchronization between distal neural sources was computed on contour and non-contour trials by the PLV (Lachaux et al., 2000) between all pairs of neural sources and frequency intervals obtained by DWT, resulting in *N*(*N* − 1) × *f* phase synchronization values for each behavioral condition (contour and non-contour) where *N* corresponds to the total number of neural sources, and *f* corresponds to the different frequency intervals. As the number of trials influences the PLV estimation, the PLV was computed on 90 trials, selected randomly from the available set of trials per subject. To compare PLV across behavioral conditions we performed a cluster-based permutation test where only significant PLV differences are kept for further study (*p* < 0.01; see Maris and Oostenveld, 2007). In short, t-statistics of a phase difference (a pair of neural sources, on a frequency *f*) were clustered according to functional adjacency between neural sources, or in other words, we analyze the PLV between particular subsets of neural sources. Here, we analyzed PLV between the neural sources PCO-FCO (see Table 1) and the PLV between PCO and frontal neural sources that are non-FCO (see Supplementary Materials for details on how we obtain the neural sources that lie within a cortical area of interest). The PLV-t-statistics were accumulated over neural sources (cumulative sum) so that the value of the t-statistics could only be positive/negative if, over all PLV pairs considered, there was a significant positive/negative t-statistics. For example, the PLV of the PCO neural sources at theta frequency (**Figure 4**) for contours and non-contour trials, was significantly different for 32 connections to FCO neural sources, so that if the visualized t-statistic was positive/negative for a certain time point, it has a significance *p*-value of *p*^(32)^. To test whether the difference between contour and non-contour trials arose due to the integration process itself, we estimated whether the t-statistics was significantly different relative to its baseline with z-score (see Supplementary Materials).

**Table 1:** **Coordinates of the cluster centroids in Talariach space and their spatial localization (Lancaster et al., 2000)**.

**Cluster name**	**Reference on the text**	***x, y, z* Talariach coordinates**	**Anatomical structure**	**Brodmann area**
OVS	Occipital cluster at visual stimulation onset	(4.44, −66.47, 26.87)	Occipital lobe precuneus	Brodmann area 31
PCO	Parietal cluster at contour onset	(−10.47, −57.19, 22.89)	Parietal lobe precuneus	Brodmann area 31
FCO	Frontal cluster at contour onset	(1.85, 35.98, 3.96)	Frontal lobe medial frontal gyrus	Brodmann area 32
OCO	Occipital cluster at contour onset	(−10.99, −76.39, 20.40)	Occipital lobe cuneus	Brodmann area 18
FRC	Frontal cluster at response cue onset	(8.02, 32.06, −17.8)	Frontal lobe medial frontal gyrus	Brodmann area 11
PRC	Parietal cluster at response cue onset	(4.40, −60.69, 14.7)	Parietal lobe posterior cingulate	Broaddman area 23
FNC	Frontal cluster not related with FCO	(−1.47, 47.84, 11.39)	Frontal lobe medial frontal gyrus	Brodmann area 10

## Results

### Behavioral analysis and neural source reconstruction

Electroencephalographic activity (EEG) and eye movements were recorded from 15 participants who were instructed to identify two different orientations of an oval-like contour (up/down), pairing the possible spatial orientations with a saccadic response in a two-alternative forced choice (2AFC) paradigm (see Figure 1). Participants correctly identified the pointing direction of contours on 97.9% of trials when a contour was present. Further analysis revealed no performance differences between trials where the contours appeared on the left/right hemifield, on five different positions in regards to the horizon, and at the three different time onsets in regards to the start of the trial (all with *p* > 0.2).

To this end, several aspects of the behavioral task can be discussed. Firstly, as the presence of spatial cues may lead to an asymmetrical shift of attention before the contour appeared (Summerfield and Egner, 2009), contours appeared on 50% of the trials (on either left/right hemifield, on five different positions in regards of the horizon, see Supplementary Materials), so that the appearance of the contour could not be predicted, as confirmed by the lack of biases on the contour categorization performance. Secondly, as eye movements introduce both amplitude changes on the amplitude of the EEG signal and a broadband increase in gamma oscillatory activity (~30–100 Hz) (Yuval-Greenberg et al., 2008; Plöchl et al., 2012), subjects were required to maintain fixation until the response cue onset, where they had to report contour identity. Additionally, and to avoid a temporal association between contour and saccadic response (Badler and Heinen, 2006), the response cue appears after a delay period of either 1.03, 1.50, or 1.97 s, once the co-aligned contour disappears. Third, subjects were required to perform an up/down eye movement at every trial (including non-contour trials), so that the trials cannot be solely distinguished based on the planning and execution of an eye movement. Fourth, note that due to their size (~11° of visual field), the contours could not be detected by individual receptive fields, but rather required the integration of activity across multiple cortical columns (Field et al., 1993; Hess et al., 2001; Mathes et al., 2006). Finally, as contours were continuously morphing, the time at which they were identified typically varied from trial to trial. To account for this variability, we computed the time interval at which the contour is identified with a probability above chance, what we call the range of contour visibility (±66 ms around the CO event). Furthermore, the CO corresponds to the point in time at which subjects identify contour with a probability of 0.70 on average (see Materials and Methods). Finally, through this study we will analyze neural signals associated to three different behavioral events (see Figure 1): VS, visual stimulation onset; CO, contour onset (contour and non-contour trials); and RC, response cue onset.

One of the core issues when measuring oscillatory activity within EEG/MEG is the difficulty of attributing scalp signals to the activation of a particular area at cortical level. As a result, synchronization between neural signals across distal locations may reflect the leaking of a single source to several electrodes or several electrodes reflecting a single source (Kujala et al., 2008; Hipp et al., 2011). Here, to improve the spatial specificity of our data and analyze oscillatory activity with higher signal to noise ratio, we performed ICA on the raw EEG data (see Materials and Methods and Table 1).

### Local synchronization during visual stimulus processing and contour processing

We started by analyzing the neural sources that are generally associated with the processing of visual stimuli. The neural sources of interest were identified though a new analysis approach which selects neural sources based on their ability to predict the behavioral condition of interest, and does not require a pre-selection of recording/analysis sites. The method is based on the hypothesis that the oscillatory activity of neural sources can predict subject perception (see Materials and Methods). In short, the time-frequency decomposition of a neural source is used as a predictor variable in a logistic regression model, such that, for each neural source, we can estimate the probability of a neural source to participate in a behavioral event of interest in a time-resolved fashion, which we call the prediction trace (Figure 2A for an example). In other words, the higher the prediction trace, the stronger the association of the neural source to the behavioral event of interest (e.g., contour lateralization). Only neural sources that statistically significantly predicted the behavioral condition of interest were kept for further study (with *p* < 0.01 based on cluster-based permutation testing, see Materials and Methods). With this process, we obtain a prediction trace for each neural source of each subject. To this end, we clustered the neural sources based on their predictive power (k-means clustering of prediction traces), thus obtaining a pooled prediction trace that reflects the probability of a group of neural sources, across subjects, to predict a behavioral event of interest.

Across all subjects, we found a total of 35 neural sources to be associated with the processing of visual stimuli or, in other words, oscillatory activity within 35 neural sources can be used to predict that a subject is processing visual stimuli on a single trial basis (an average of 2.3 neural sources per subject).These neural sources predicted the presence of a visual stimulus in 87.2% of the trials on average, at 100 ms after visual stimulation onset (VS onset), with time-varying prediction traces that were similar across subjects and across neural sources (Figure 2A left). Interestingly, the dipole locations of these sources spread across large areas within the occipital cortex (see Table 1 for the centroid coordinates of the cluster OVS, and Figure 2B left), in accordance with a large body of studies that argue for the involvement of occipital areas on the processing of visual information (Hubel and Wiesel, 1962; Goodale and Milner, 1992; Tallon-Baudry and Bertrand, 1999). For the sake of completeness, we represented the oscillatory activity of these neural sources in form of a spectrogram (Figure 2C), showing that visual stimulus onset induces an enhancement of gamma band synchronization that extends over a broad frequency range (30–100 Hz) accompanied with a slight enhancement of oscillatory activity in the low frequency bands (5–30 Hz) within the first 500 ms after VS onset. Taken together, this findings replicate the well-known neural signature associated with the processing of visual stimuli involving the occipital cortex, as reported in human MEG/EEG studies (Lutzenberger et al., 1995; Donner et al., 2007; Volberg et al., 2013) and invasive recordings (Gray et al., 1989; Li et al., 2006), confirming that our analysis method, coupled with ICA source decomposition and the localization of these sources, allows for the reconstruction of local population activity associated with a given behavioral condition of interest.

Next, we analyzed neural activity that associate with contour processing, namely, we identified those neural sources that are predictors of correct contour categorization at CO (see Figure 1). Across all subjects we found a total of 31 neural to associate with contour categorization (i.e., average of 2.06 neural sources per subject). Clustering these neural sources based on their prediction traces gave rise to two distinct groups (k-means clustering, see Materials and Methods). The first cluster (PCO) is predictive of contour perception within 50–00 ms, with a correct prediction average of 65.78% trials at 380 ms after CO (±66 ms range of contour visibility Figure 2A, right), a comparable performance to other decoding approaches (Rotermund et al., 2011). Dipole localization shows that these neural sources are mainly grouped within parietal cortex (see Table 1 for the centroid coordinates and Figure 2B, right). Concurrently, the second cluster (FCO), is localized within frontal areas (see Table 1 for the centroid coordinates and Figure 2B, right), and is predictive of contour perception for the first 500 ms after CO, with an average correct prediction in 64.26% of trials at 300 ms after CO (Figure 2A, right). The spectral decomposition of parietal and frontal neural sources associated with correct contour categorization (cluster PCO and FCO; Figure 2D, upper and lower, respectively) shows enhanced oscillatory activity within the theta band (4–8 Hz), co-occurring with a broadband enhancement of gamma oscillatory activity (>30 Hz), especially within low-gamma frequency bands (30–60 Hz). Simultaneously, while beta oscillatory activity (13–30 Hz) seems to be slightly reduced in parietal neural sources, a strong and long-lasting enhancement of beta is present within frontal areas (cluster FCO; Figure 2D, lower).

In summary, we have shown that oscillatory activity within parietal/frontal cortices in single trials can be used to predict the subject's ability to correctly classify contours, revealing a crucial involvement of higher visual cortices on the perceptual grouping of local elements onto a coherent percept.

### Dissociating the processing of contours for categorization from the contour linking process and saccadic control

As perceptual grouping through contour categorization seem to require the processing of contours within parietal/frontal cortices, we continue by testing whether we can disentangle this process from the process of linking the contour elements. In particular, we tested whether oscillatory activity can predict the spatial location of the contour or, in other words, whether the contour appears on the left or right hemifield at a given trial (Contour Lateralization test). The spatial localization of the contour is not a relevant feature for the behavioral task, and we cannot disentangle whether the contour is perceptually available at this stage. Thus, if we can decode the contour spatial location, it is necessarily required that the contour elements have already been linked into a whole.

Oscillatory activity within parietal/frontal neural sources associated with contour processing is assumed to reflect neural processes involved with the processing of contours for further categorization, which may involve top-down attentional selection (Siegel et al., 2008; Van Ede et al., 2012), memory matching (Herrmann et al., 2004) or the targeting of the contour (VanRullen and Thorpe, 2001; Sato and Schall, 2003). However, broadband gamma synchronization within parietal cortex has been proposed to encode motor goals within visuo-motor tasks (Van Der Werf et al., 2008), and enhanced gamma activity within frontal cortex has been associated with eye-motor control, encoding saccadic goals (Schall and Thompson, 1999). To test whether the oscillatory activity within parietal/frontal neural sources arises due to the processing of contours for further categorization or to saccadic control, we tested whether neural sources can predict the saccadic goal, a process associated with saccade planning and execution (Saccade Planning test).

**Figure 3:**
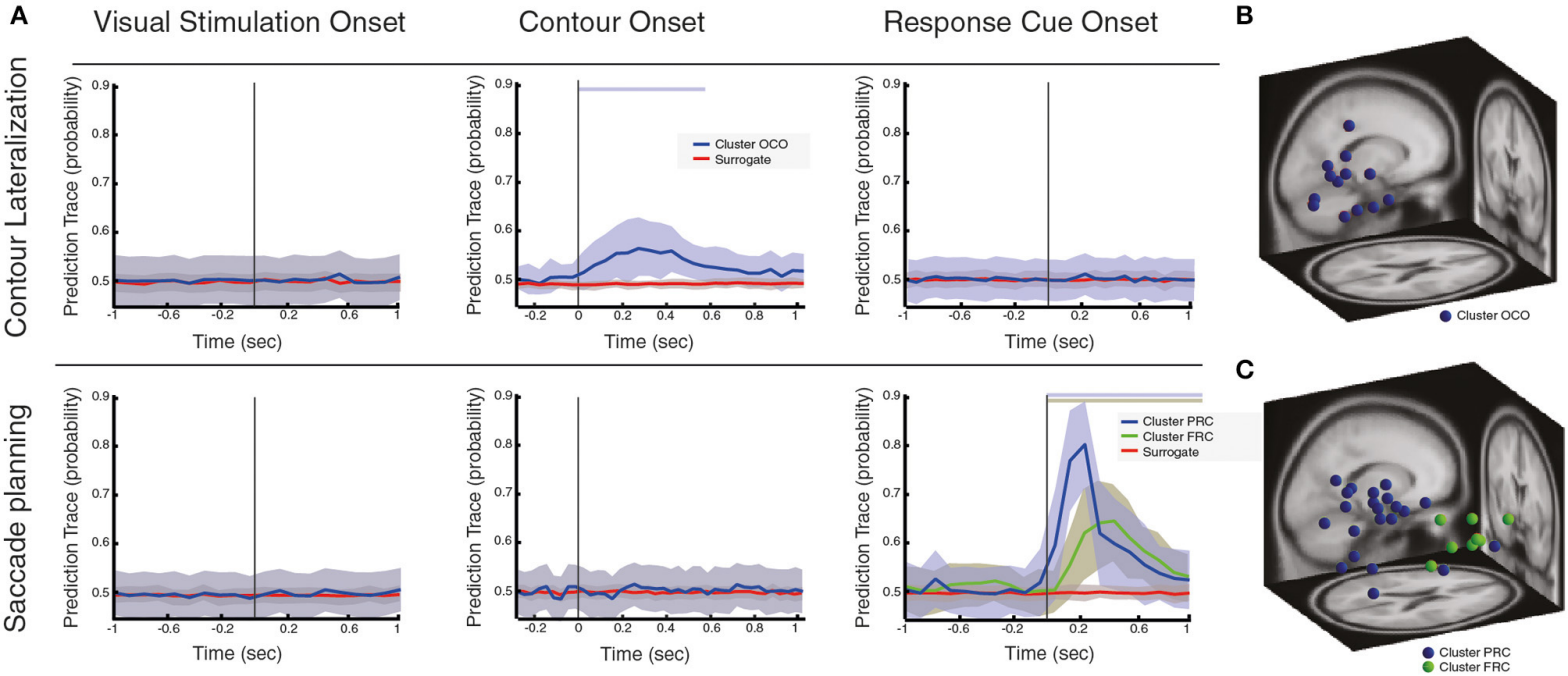
**Local oscillatory activity during contour linking process and saccadic control**. **(A)** Prediction traces at three different behavioral events (i.e., VS, visual stimulation; CO, contour onset; RC, response cue), for two different conditions of interest: contour lateralization and saccade planning. Prediction traces of neural sources across subjects are shown in blue/green (mean ± std), while prediction traces of the surrogate data is shown in red (mean ± std). Significance of prediction traces is shown as a shadow in upper part of the plot, colorcoded for different clusters (*p* < 0.01, corrected for multiple comparison). **(B)** Dipole localization of neural sources associated with contour lateralization within CO onset. **(C)** Dipole localization of neural sources associated with saccade planning within RC onset.

Following the same methodology as in the previous section, we estimated the prediction trace of neural sources associated with each of the three different behavioral events (i.e., VS, visual stimulation onset; CO, contour onset; RC, response cue onset), and identified potential neural sources that are predictors of contour lateralization or saccade planning, see Figure 3A. As such, neural activations within VS are used as a double negative control, since they should not associate with either saccade planning or contour lateralization. Alternatively, we would expect that some neural sources within CO and RC may be predictors of saccade planning.

First, our results show that neural sources that respond to VS do neither predict saccade planning nor contour lateralization (Figure 3). Second, we found that neural sources that are active during the CO event are not found to be associated with saccade planning, while a subset of neural sources does predict contour lateralization (cluster name OCO, Figure 3A, total of 14 sources, with an average of 0.99 neural sources per subject). At 250 ms after CO (±66 ms range of contour visibility), the spatial localization of the contour within the visual field can be predicted with an average accuracy of 57.7% (significantly different from chance level, *p* < 0.01). Interestingly, dipoles of the neural sources that predict contour lateralization are localized within the occipital lobe and are independent of those neural sources that associate with contour categorization (see Table 1 for the centroid coordinates and Figure 3B). Furthermore, the prediction trace of contour lateralization within higher occipital areas peaks 100 ms earlier than the prediction trace of parietal neural sources associated to contour categorization, FCO. Third, oscillatory activity of neural sources at RC onset were not associated with contour lateralization, suggesting that low-level stimulus properties are not maintained up until the RC event (Figure 3A). In contrast, a subset of 32 neural sources (total across subjects, an average of 2.13 neural sources per subject) are associated with saccadic direction, which are separated into two clusters according to its prediction traces: PRC and FRC (Figure 3C). Both clusters are predictive of saccadic direction within the first 600 ms after RC onset, with a peak prediction of 85.78 and 74.38% accuracy after 200 and 300 ms after RC, respectively. Dipole localization of the neural sources associated with saccadic planning is presented in Figure 3C and Table 1. Interestingly, the neural sources of cluster PRC localize within the parietal areas while cluster FRC localize within the frontal cortex. In accordance with recent studies on saccadic control, our results show that both frontal and parietal cortex are involved in saccade preparation and execution (Schall and Thompson, 1999; Sato and Schall, 2003; Van Der Werf et al., 2008). Notably, the set of neural sources within parietal and frontal cortex that associate with saccadic control (PRC and FRC) is not overlapping with the set of parietal and frontal sources that associate with contour integration (PCO and FCO).

In summary, neural sources that are associated with the processing and manipulation of contours to be correctly classified (PCO and FCO) are dissociated from the linking process of local elements into a whole, which can be decoded from oscillatory activity within occipital areas. Furthermore, the neural sources that associated with the overall perceptual grouping process are dissociated from complementary processes that are present during visual processing (e.g., saccade planning). Interestingly, while several behavioral processes can be decoded from neighboring spatial locations (contour categorization and saccadic control), they appear to be segregated neural processes in both time and IC-space.

### Long-range networks of oscillatory activity during contour processing

To ascertain whether contour processing and categorization manifests itself within the global synchronization network, we quantified transient phase-synchronization across distant neural sources (Lachaux et al., 2000) for five different frequencies: theta (5.6 Hz), alpha (11.16 Hz), high beta (22.32 Hz), low gamma (44.6 Hz), and high gamma (89.3 Hz) bands (main frequency within frequency interval, see Materials and Methods).

We first addressed the question of whether the transient synchronization pattern arising during contour trials where the contour is correctly classified differs from the synchronization pattern of non-contour trials. For that, we estimated whether the PLV significantly differs between the two conditions (contour vs. non-contour) by means of a permutation test. The 21.5% of all neural sources show statistically significant synchronization at all frequencies tested (*p* < 0.01), suggesting that there is a dynamic formation of phase-synchronization networks across distal neural sources during cognitive processing (further details in Figure S3).

**Figure 4:**
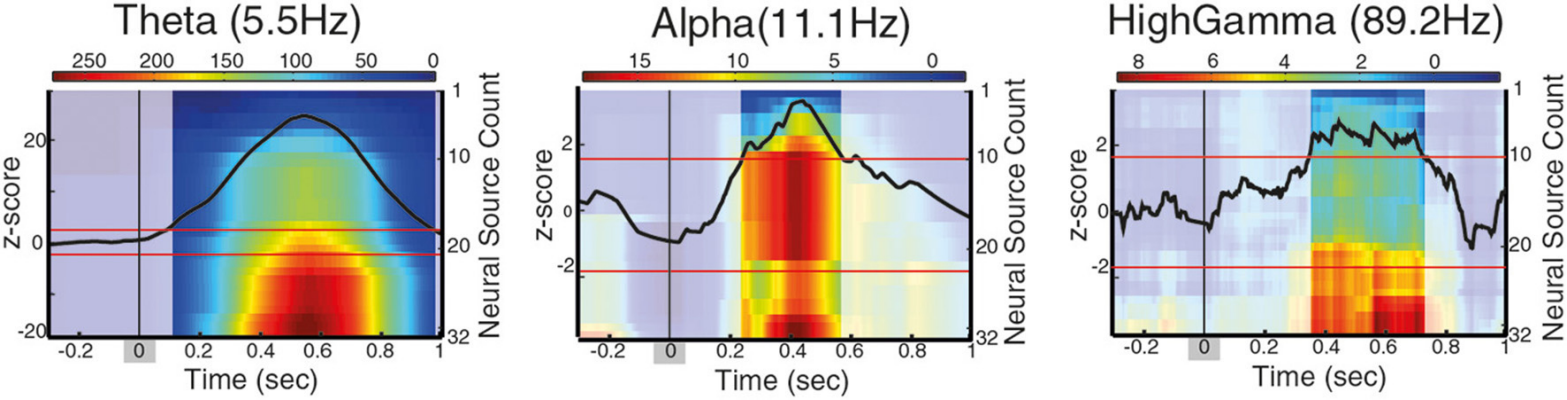
**Long-range phase synchronization during contour integration between PCO/FCO neural sources, for different frequency intervals**. The images show the cumulative sum of the t-statistics for the Phase Locking Value difference during contour integration process (contour onset at *t* = 0 ± 66 ms for the range of contour visibility, marked as a shadow gray). Intense color indicates periods of significantly enhanced phase synchronization with distant neural sources (positive t-statistic), corrected for baseline by z-score. A black line indicates the z-score of the neural sources associated with contour integration, red lines indicate z-score significance threshold.

So far, long-range synchronization between distal neural sources has been identified by grouping neural sources according to their spatial proximity (Palva et al., 2005; Hipp et al., 2011). However, as spatially neighboring neural sources may be involved in more than a single behavioral function, we focus on the properties of long-range synchronization of distal neural sources based on their involvement with the behavioral task of interest rather than on their spatial location. Specifically, we addressed the question of how the neural sources associated with contour processing modulate long-range synchronization networks. For that, we analyzed the connectivity pattern of parietal/frontal areas associated with contour categorization (PCO-FCO) and found that phase-synchronization in contour trials is significantly higher than in non-contour trials at theta between 80 and 985 ms after CO, at alpha within 245–570 ms after CO, and at high gamma between 350 and 720 ms after CO (±66 ms range of contour visibility for all measures, Figure 4). In principle, the differences in phase-synchronization between contour and non-contour trials may either reflect neural processes directly related to the perceptual grouping or to secondary processes (e.g., saccade preparation). To differentiate the two possibilities, the significance of t-stats is corrected by the z-score, which estimates whether the t-statistics is significantly different when compared to its baseline (z-score, see Supplementary Methods).

Previous studies report phase locking between frontal and parietal areas while performing visual processing tasks at the beta frequency range (Palva et al., 2005; Phillips et al., 2012), while our analysis found synchronization at theta, alpha, and gamma frequencies. Since our results suggested that different functions can occur in overlapping brain areas, we hypothesize that the coupling between parietal/frontal areas in beta frequency may not be required for the recruitment of neural sources that are enhanced on a behavioral task, but may instead signal a baseline communication or status quo, as proposed by the beta suppression hypothesis (Engel and Fries, 2010; Schroeder et al., 2011). To test this hypothesis, we analyzed the phase-synchronization pattern of PCO neural sources to frontal sources that are not related to contour processing (cluster FNC, see Table 1 and see Supplementary Materials for details), and found that phase-synchronization of these sources is significantly different between contour and non-contour trials in the theta range (5.5 Hz), starting 80 ms after stimulus onset, at alpha (11 Hz) within 250–550 ms and at high beta (22.6 Hz) within 280–440 ms (±66 ms range of contour visibility for all measures). As such, our results suggest that gamma synchronization between parietal and frontal sources is functionally related to contour processing, while beta synchronization between parietal and frontal non-contour-integration specific sources might reflect secondary processes but not contour processing and categorization itself.

Finally, to further explore the relevance of long-range connections between distal neural sources, we tested whether there is a significantly different number of connections between different sets of neural sources (number of links to other neural sources with significant PLV). We found that parietal sources associated to contour processing -PCO- have a significantly higher number of connections than frontal neural sources -FCO- for any of the tested frequency intervals (Figure S3), suggesting that parietal neural sources may form a hub-like structure underlying coordination of distal neural sources during visual perception. Interestingly, we did not found a significant increase of the number of connections between PCO-FCO, suggesting that phase-synchronization coupling exist but it is not significantly higher than the connectivity that can occur between two random neural sources.

Taken together, these results suggest that the parietal/frontal areas associated with contour processing (PCO/FCO) show enhanced phase synchronization in theta, alpha and high gamma frequency that arises in trials where the contour is perceptually grouped and classified, while beta synchronization is found between parietal and frontal but non-contour sources (PCO/FNC). Notably, the neural sources localized in the parietal areas and associated to contour processing (PCO) exhibit higher connectivity to distal cortical areas as compared to frontal sources (FCO), therefore forming a hub-like structure underlying the coordination of neural processes involved in contour processing.

## Discussion

In this study, we aimed to first determine whether oscillatory activity within early-visual cortical areas predict the perceptual grouping of a visual stimuli; and second, we determined whether this perception process modulates long-range synchronization networks.

To answer those questions, we analyzed EEG activity of subjects performing a contour categorization task, where perceptual grouping can be described as a two-stage process (Roelfsema, 2006; Watt et al., 2008; Mack and Palmeri, 2010; Kourtzi and Connor, 2011; Volberg and Greenlee, 2014) as the successful execution of the task requires the linking of contour boundaries into a coherent contour (Contour Linking Process), as well as a processing of the contour for its correct categorization (Contour Processing). Contour integration follows the Gestalt law of “good continuation” and has been serving as a reference behavioral task to study visual perception as local stimulus features remain constant, thus minimizing variability due to low level stimulus features (Wertheimer, 1923; Field et al., 1993; Hess et al., 2001; Mathes et al., 2006). In other words, given that contour and non-contour stimuli only differ on the co-alignment of a small subset of elements, differences in the oscillatory activity within the neural sources reflect differences between perceptual states. While psychological, psychophysical and neuroimaging studies propose that local interactions within early visual cortex mediate contour integration (Field et al., 1993; Li et al., 2006; Mathes et al., 2006), recent studies report that contour detection and its neural signals can be modulated by the task requirements, including attentional demands (Roelfsema et al., 2004), perceptual learning (Li et al., 2006) or perceptual noise within the contour (Mathes et al., 2006). Here, through the analysis of the EEG signals with a pattern classifier, we decode both the contour linking process as well as the processing of the contour for its correct categorization from a contour categorization task, with no pre-selection of the cortical areas of interest. Furthermore, classical contour integration tasks involve the sudden appearance of a contour and its background elements, so that the contour appearance is inevitable linked to a sudden change in the visual stimulus. Here, the contour integration task was adapted to mitigate the presence of spatial and temporal cues associated to the contour appearance by continuously modulating the orientation of local elements, reducing in turn the possibility of generating an asymmetrical shift of attention before the contour appeared (Summerfield and Egner, 2009, see Video 1 for a contour trial). Notably, the dynamic design allows, for the first time, the dissociation of the neural signatures associated with the onset of the visual stimulus and the appearance of a contour.

Accordingly, in the first part of the study, we aimed to determine whether local oscillatory activity can predict perceptual grouping of a visual stimuli, involving both the linking of local elements into a contour structure (Contour Linking Process) and the further contour processing for its categorization (Contour Processing), which may involve a broad range of secondary processes, such as top-down attentional selection (Siegel et al., 2008; Van Ede et al., 2012), memory matching (Herrmann et al., 2004) or the targeting of the contour (VanRullen and Thorpe, 2001). Our results show that oscillatory activity within occipital cortex allows for the decoding of the spatial location of the contour, indicating that at this stage, the local elements that form the contour are linked into a coherent structure, supporting the idea that occipital areas are classically linked to the processing of visual stimulus in a bottom-up manner (Hubel and Wiesel, 1962; Gross, 1999). Most interesting for this study, oscillatory activity within the frontal and parietal cortex can predict correct categorization of the contour, in line with the idea that top-down control is involved in perceptual grouping (Li et al., 2006; Mathes et al., 2006; Volberg et al., 2013). Whereas occipital sources that reflect the linking of local elements peak at 250 ± 66 ms after CO, frontal cortices better predict contour categorization at 300 ± 66 ms, followed by the parietal neural sources that peak at 380 ± 66 ms, suggesting a dynamics of contour categorization resembling visual search tasks (Buschman and Miller, 2007; Phillips et al., 2012). Our study advocate for a crucial involvement of fronto-parietal areas on a perceptual grouping task that requires contour categorization, areas that are proposed to mediate the formation and selection of behaviorally relevant stimulus (Sato and Schall, 2003), as well as attentional control (Mesulam, 1999; Siegel et al., 2008). Furthermore, our results emphasize the relevance of local oscillatory activity and suggest that enhancement of local synchronization within cortical areas serves as a general mechanism mediating sensory processing (Singer, 1999; Fries, 2009; Hipp et al., 2011). Finally, and most important for the purpose of this study, our results show that several aspects of a behavioral task (e.g., contour categorization and saccade planning) can be decoded within nearby spatial locations (e.g., parietal and frontal cortices). To this end, we argue for the advantage of using pattern classifiers for the analysis of time-resolved brain activity, proposing that this approach increases sensitivity on studying the neural basis of cognitive processes.

The second part of the study aimed to determine whether the integration and categorization of contours manifests itself as a transient synchronization involving distal neural sources. Though the understanding of how cognitive functions modulate synchronization across distributed cortical populations have greatly improved, the measure of such synchronization from EEG/MEG recordings remains difficult. This is mostly due to methodological issues. First, the low spatial resolution of EEG complicates both the cortical localization of neural activations and the computation of long-range synchronization (Kujala et al., 2008; Siegel et al., 2012). To account for this problem, we analyzed EEG signals at the source level, a transformation of electrode data into localized cortical sources, which increased spatial specificity. Secondly, there is a clear lack of statistical tools that allows the analysis of high-dimensional neural signals with no prior assumptions on the structure and the location of the neural signals associated with the behavioral process of interest (Kilner, 2013). Our method to detect neural signals associated with whichever behavioral conditions of interest may serve as a powerful new tool to analyze high-dimensional neural data, where the selection of neural signals of interest is hypothesis free, with no starting assumptions on functional specialization and localization of neural sources. Instead, the method provides quantification for how well a neural signal predicts the behavioral condition of interest, simultaneously increasing the signal to noise ratio by selection of relevant neural signals. Furthermore, the method corrects for multiple comparisons and in principle, it is not limited to the study of EEG signals, but can be applied to any time-varying signal associated with categorical conditions. As such, our approach complements recent brain-computer applications that quantify structural properties of neural processes (Nicolelis and Lebedev, 2009; Rotermund et al., 2011; King and Dehaene, 2014). Note that the method is constrained to the analysis of neural signals associated with categorical behavioral conditions, where the model itself assumes that the relationship between the behavior and the, potentially non-linear, predictors is linear.

Our analysis show that 21.5% of the neural sources show an intermittent phase synchronization with other neural sources while performing the contour categorization task, supporting recent studies which suggest that there is a dynamic control of information flow across distributed neural sources in a frequency-specific fashion (Engel et al., 2001; Palva et al., 2005; Hipp et al., 2012). Is there a long-range synchronization network specifically associated to the processing of contour percepts for further categorization? For that, we analyzed the phase synchronization between neural sources that are associated to predict the subject's ability to classify contours (clusters PCO/FCO) and found that they synchronize at theta (5.6 Hz, main frequency within interval), alpha (11.6 Hz) and high gamma (89.3 Hz) frequency intervals. Those fluctuations seem to mediate information transmission between parietal/frontal areas which are specifically involved to the processing and manipulation of a contour for its categorization, in congruency with previous studies that report transient synchrony in the high-gamma band to emerge during perceptual binding (Melloni et al., 2007; Phillips et al., 2012), cross-modal integration (Hipp et al., 2012) and attentional control (Mesulam, 1999; Siegel et al., 2008), proposing that task-relevant cortico-cortico communication from between cortical areas may be mediated through gamma synchronization (Fries, 2009). Strikingly, beta synchronization between parietal/frontal areas has been reported in visual processing tasks, such as visual working memory, visual search and visual attention studies, suggesting that long-range beta synchronization may mediate top-down communication between cortical areas are active in tasks involving visual information processing (Munk et al., 2002; Engel and Fries, 2010; Hipp et al., 2011; Morgan et al., 2013; Volberg and Greenlee, 2014). Further, we analyzed phase synchronization between parietal and frontal neural sources that are not associated with contour processing (cluster FNC) and found that they indeed show phase synchronization in the beta frequency range (22.3 Hz). As such, while distal neural sources actively involved in the processing of contours synchronize at gamma frequency, nearby frontal sources synchronize in beta frequency. As observed in studies within the motor control system, where beta is actually replaced by gamma-band oscillatory activity during the preparation and execution of voluntary movements (Pogosyan et al., 2009; Swann et al., 2009), beta synchronization between parietal/frontal sources between neural sources that are recruited for a particular cognitive task at hand may similarly signal the maintenance of baseline activity, facilitating the cross-modal integration of cognitive tasks, allowing for the processing information on different timescales (Engel and Fries, 2010; Schroeder et al., 2011). These results seem to provide further insights on how nearby cortical sources enhance oscillatory in different frequencies in a task-specific manner, emphasizing the relevance on analyzing neural activity based on function rather than analyzing neural activity based on its spatial location.

Taken together, our results suggest that while oscillatory activity within occipital cortex predict the linking of local elements into a contour, oscillatory activity within parietal and frontal cortices play a crucial role in the execution of a contour categorization task, as well as the establishment of transient synchronization among them. In particular, our study reveals a phase locking in alpha, theta and gamma frequencies between frontal and parietal neural sources arising during the correct contour categorization, while a fronto-parietal beta phase locking arises within those neural sources that are not actively recruited in the contour categorization task itself. Finally, we presented a novel method that identifies neural sources based on their ability to predict behavioral conditions of interest, and report that different behavioral functions may involve the activation of cortical areas within nearby spatial locations, suggesting the presence of functionally distinct but spatially overlapping cortical areas.

### Conflict of interest statement

The authors declare that the research was conducted in the absence of any commercial or financial relationships that could be construed as a potential conflict of interest.
