# Supplementary material for: Neuronal oscillations form parietal/frontal networks during contour integration
Source: Front Integr Neurosci. 2014 Aug 13;8:64. doi: 10.3389/fnint.2014.00064 (PMC4131516; doi:10.3389/fnint.2014.00064)
Supplement: Supplementary file 1 [file DataSheet1.PDF]

Supplementary Material

Neuronal Oscillations during contour integration of dynamic visual stimuli form parietal/frontal networks

Marta Castellano[1*], Michael Plöchl[1], Raul Vicente[2], and Gordon Pipa[1]

1. Institute of Cognitive Sciences, University of Osnabrück, Germany. Address: Albrechtstrasse 31, 49076 Osnabrück

2. University of Tartu, Faculty of Mathematics and Computer Science, Institute of Computer Science. Address: Juhan Liivi 2, 50409 Tartu, Estonia

[*]**Correspondence**: Marta Castellano. Institute of Cognitive Sciences, University of Osnabrück, Germany. Address: Albrechtstrasse 31, 49076 Osnabrück.

m@martacastellano.eu

1. Supplementary Materials and Methods

1.1 Participants

Eighteen healthy participants (11 female and 7 male, aged 18-32 years) gave informed consent to be involved in the experiment. All participants reported normal or corrected-to-normal vision, with no history of neurological or psychiatric illness. Three subjects had to be excluded from the analyses due to either a malfunction of the recording system, extremely low number of trials after preprocessing, or a chance level performance on the behavioral task. As such, a total of 15 subjects were analyzed in this study. The study was approved by the local ethics committee and conducted in accordance with the Declaration of Helsinki and national guidelines. Participants received either monetary compensation or course credits usable at the University of Osnabrück.

1.2 Visual stimulation: generation of visual stimulus

At each trial, participants were presented with a frame of randomly oriented Gabor elements with a continuously changing orientation, leading to the perception of smoothly rotating Gabor elements through the trial (see Video 1 for a contour trial and Video 2 for a non-contour trial, .avi format). The modulation of the angle of Gabor elements introduces temporal evolution on the contour formation, so that collinear contours are continuously morphing and perceived as dynamic stimuli with smooth progression between stimulation frames.

A stimulation frame consists of 335 randomly oriented Gabor elements, on average (each spanning 17 pixels or 0.5º visual angle, see Figure S1 A), distributed pseudo-randomly on the viewing field (similar to Mathes et al., 2006). A subset of 20 Gabor were placed on the contour path, an oval-like shape that extends approximately 11.3º of visual field. As such, a contour is defined as a set of co-aligned Gabor elements to the contour path, where co-alignment or degree of co-alignment φ represents the distribution of angles diverging from the optimal

angle given by the tangent of the contour path (a degree of co-alignment of φ=0 indicates that all Gabor elements are co-aligned to the contour path). Contours appeared in either left or right hemifield on five different positions relative to the horizon (see Figure S1 C).

The contour was chosen to be an oval-like shape such that the curvature at both asymmetric ends was rather similar. This required the subjects to integrate most of the contour to be able to identify the pointing direction of the oval. Pointing direction of the oval is the direction at which the sharp side of the oval is pointing to. The contour could either point up/down the screen, the two classes that subjects had to identify.

To control whether the contour could be identified by the spatial location of the Gabor elements, we computed the distance across nearest neighbors Gabor elements within the contour (Euclidean norm), within non-contour (distracters), and within all Gabor elements (see Figure S1 B). This analysis showed that there is no difference on the distance of Gabor elements whether they are located within contour or not. An example of an stimulus frame that contains a contour is presented in Figure S1 D, where instead of co-aligned Gabor elements, the location of the elements is marked by a star.

The orientation of all the Gabor elements changes with 2 ± 2.6 (mean ± std) angle per frame. This temporal modulation of the orientation of Gabor elements can be seen as a diffusion process, where the orientation of Gabor elements changes over time. The diffusion process that leads to co-aligned contour from a field of randomly oriented Gabor elements was generated as follows: the first stimulus frame to be generated is the one that contains a subset Gabor elements co-aligned to the contour path, while the distracters have random orientations uniformly distributed between [0, 2pi]. To undo this structure (co-aligned Gabor elements morphing to a field of randomly oriented Gabor elements), the orientation of each Gabor element is changed by a diffusion process, so that the orientation at the next frame $F(t+1)$ is the orientation at the current frame $F(t)$ plus $n(t)$, where $n(t)$ is a random number normal distributed with mean 2 and standard deviation 2.6. As such, within successive frameworks the co-aligned contour structure will diffuse until the orientation of all Gabor have a similar variability and are indistinguishable from uniformly random orientated Gabor elements. To generate co-aligned contour structure from a random field of Gabor elements, the diffusion process is played back in time. Hence, the first frames shown to the subject were frames with the largest variability of Gabor orientation. An example of how the orientation of Gabor elements changes over time is provided in Figure S1 E. The degree of co-alignment φ is marked as a thick line ± standard deviation (circular standard deviation, dotted line). The dark vertical lines mark the time point at which contour co-aligns at either 1.02, 1.50 or 1.97 s (contour onset event). The shadowed area indicates the range of contour visibility of the contour (see section 'range of contour visibility'). The changes in the direction of movement were implemented by inverting the sign of the mean.

Visual stimulation was displayed on a 23 inch DELL-U3211HB monitor (VGA mode, 1024 x 768 pixel resolution, refresh rate 60Hz). Effectively, each stimulus frame stayed 16.7 ms on screen. The participants viewed the screen binocularly at 60 cm distance in a room with dim light and constant luminance.

1.3 Behavioral task

Participants were asked to respond to 720 trials of a two-alternative forced choice visual identification task. As such, participants were instructed to identify two different orientations of the same contour, pairing each of the possible spatial orientation of the contour with a

saccadic response (see Figure 1). Trials were divided into several experimental blocks of 40 trials each, with voluntary break periods.

Each trial starts with the appearance of a fixation dot (size 0.3º visual angle, see Figure 1), lasting for 300 ms. Visual stimulation starts with a field of randomly oriented Gabor elements and lasts either 1.03, 1.50 or 1.97 s (see section range of contour visibility onset).This is the first behavioral event considered for analysis on the paper: visual stimulation onset or VS event.

After this delay period, a subset of local elements would co-align its orientation to the contour path. Contours appeared in 50% of the trials, on either the left or right hemifield (25% of total trials) on five different positions relative to the horizon (see Figure S1 C). This is the second behavioral event considered for analysis: the contour integration onset or CO event, which has two behavioral conditions associated: contour versus non-contour trials. After a delay of 1.033 s, Gabor elements involved in contour loose co-alignment to the contour path.

After a delay period lasting either 1.03, 1.50 or 1.97 s (see section range of contour visibility onset), the report cue appears (RC event), indicating the moment when participants can report perception of the contour. Participants are instructed to report where the contour is pointing by saccadic movement towards the target location. The target location is marked by a black rectangle located either up/down of the fixation the stimulus frame (see Figure 1 and visual stimulation section).

The delays period before and after the appearance of contour are balanced so that the total trial length adds up to 4.033 s. As such, the subjects are exposed to three different trial classes, presented in Figure S1 E. Based on color coding in Figure S1 E, blue trials have a pre-contour delay of 1.97 s and an after-contour delay of 1.03 s, red trials present a pre-contour delay of 1.50 s and an after-contour delay of 1.50 s, and green trials have pre-contour delay of 1.03 s and an after-contour delay of 1.97 s. Figure S1 E aims to visualize the temporal evolution of the angle of Gabor elements involved in contour for the three different trials. The degree of co-alignment φ is marked as a thick line ± standard deviation (circular standard deviation, dotted line). The dark vertical lines mark the time point at which contour co-aligns at either 1.03, 1.50 or 1.97 s (contour onset event). The shadowed area indicates the range of contour visibility of the contour (see section 'range of contour visibility').

1.4 Absolute threshold of contour identification for morphing stimulus

The identification threshold of co-aligned Gabor elements has been reported to change as the degree of co-alignment between elements varies (Hess et. al, 2001). For this reason, before the EEG recordings, we performed a psychophysics experiment to estimate subjects absolute threshold, defined as the lowest degree of co-alignment that participants can tolerate to identify the contour. To compute the absolute threshold, we studied subject's performance on identifying co-aligned Gabor elements for different degrees of co-alignment φ. A total of 7 participants (independent set from those that participated in the EEG study, 5 male and 2 female, aged 24-29) gave informed consent to participate in the study, approved by the local ethics committee and conducted in accordance with the Declaration of Helsinki and national guidelines. Participants were asked to respond to 300 trials of the mentioned two-alternative forced choice visual identification task, where subjects were instructed to report contour identifications, where the contour appears 50% of the trials in either left/right hemifield (see above for further details). All the experiment parameters remained the same except for the degree of co-alignment φ (see below). Trials were divided into several experimental blocks of 40 trials each, with voluntary break periods.

The degree of co-alignment φ is varied by increasing the variability of angles diverging from the optimal angle given by the tangent of the contour path. As such, a degree of co-alignment of φ=20º indicates the addition of uniformly distributed angles between interval [-20,20] to angles that make Gabor to be co-aligned with the contour path. In other words, higher values of φ produce more divergent alignments of the Gabor elements in regard to the optimal angle given by the tangent of the contour path, leading to less defined contour and eventually to randomly oriented Gabor elements. The distribution of angle differences for different degrees of co-alignment φ tested can be visualized in Figure S2. The average subjects' performance in the contour identification task for the different degrees of co-alignment is presented in Figure S2. The detection performance of contours with a φ=0 is 97.9% (average over subjects), where a degree of co-alignment of φ=0º indicates that all Gabor elements are co-aligned to the contour path. Increasing φ to φ=40º decreases subject performance to 70%. In other words, when the contours are co-aligned to the contour path at φ=40º, the probability of a contour to be identified is 0.7. The threshold at which subjects are not able to identify the contour is φ=50º (on average), where their performance drops up to chance. In summary, the lowest degree of co-alignment of Gabor elements that lead to the identification of the contour, is φ=50º.

1.5 Range of contour visibility onset

For the main task of this study, as described above, participants were instructed to identify the two different orientations of a dynamic contour that morphed from randomly located Gabor elements. Up to this point, we should consider that, given that the contour is continuously morphing towards a co-aligned contour, the time at which the contour is identified at each trial may vary. In other words, the behavioral event of contour onset has an uncertainty associated due to its continuous morphing. We account for this uncertainty in two ways.

First, the time point at which contour co-aligns at the behavioral event 'contour integration onset' is defined as the time point in the trial at which the contour will be identified with a probability above 0.7. Second, we define the range of contour visibility, which defines the interval within the trial at which the contour will be identified with a probability above chance. The probability of a contour being identified at a time of interest can be directly estimated from the psychophysics experiment in section above. In short, the psychometric function associates the degree of co-alignment φ with a identification probability. In order to compute the probability of a contour being identified at time t, with a known degree of co-alignment φ, we inverse the psychometric function. As such, the time point at which contour co-aligns at the behavioral event 'contour onset' is defined as the time point in the trial at which the contour will be identified with a probability of 0.7. In other words, the contour integration onset time $t = 0$ marks the time point at which the contour can be identified with a probability of 0.7.

Similarly, the range of contour visibility spans ±66 ms around the contour onset event. In short, the range of contour visibility is defined as the time interval within the trial at which the contour will be identified with a probability above chance. The psychometric function obtained in the section 'Absolute threshold of contour identification for morphing stimulus', shows that with a degree of co-alignment of φ=50º, the contours are identified by chance, which corresponds to 66 ms before the contour onset event.

In summary, the behavioral event contour onset has an uncertainty associated due to its continuous morphing. To account for this uncertainty, we define as the contour onset the moment in time at which the contour will be identified with a probability of 0.7. Furthermore, we define the range of contour visibility, which defines the interval in time at which the

contour will be identified with a probability above chance, to be ±66 ms around the contour onset event.

1.6 Data recording

Synchronization of stimulus presentation, eye-tracking, and EEG recordings are controlled via ViSaGe software (Cambridge Research Systems Ltd.). Eye movements were recorded monocularly by EyeLink 1000 (SR Research Ltd.), at a sampling rate of 2000Hz. The EEG was recorded via 64 channel ActiCap (Brain Products GmbH). Electrode impedances were kept below 5 kΩ. EEG activity was amplified using a BrainAmp DC (Brain Products GmbH) system and digitalized at a sampling rate of 1000 Hz.

1.7 Analysis software

All analyses were performed in Matlab (MathWorks, Natick, MA) with custom scripts and several open-source Matlab-toolboxes: EEGLab (Delorme and Makeig, 2004) and Fieldtrip (Oostenveld et al., 2011).

1.8 Data preprocessing and artifact rejection

The raw EEG was high-pass filtered at 1Hz by a FIR filter to remove very low frequency and constant trends (provided by EEGLab, zero-phase forward-reverse). Following, EEG data was re-referenced to the average across all electrodes. Next, data was epoched into trials of 3 sec duration (-1 to 2 sec around behavioral event of interest). On a first step, trials with strong muscle activity were identified and removed by visual inspection. While this approach removed severe artifacts, we decided to reduce possible remaining artifacts by rejecting trials with extreme values, linear trends, improbable data and highly negative kurtosis were rejected (as suggested in Delorme et al., 2007). Finally, the mean baseline value was removed from each trial ([-0.5,0] sec before behavioral event of interest). Preprocessing resulted in 330±20 (mean ± std) contour trials and 210 ± 14 (mean ± std) non-contour trials per subject.

1.9 Control for microsaccade artifacts

Eye movements introduce both amplitude changes on the amplitude of the EEG signal and a broadband increase in gamma oscillatory activity (~30-100Hz) (Yuval-Greenberg et al., 2008; Plöchl et al. 2012). To mitigate presence of eye movements during EEG recording, participants are instructed to fixate during the total length of the trial. Loss of fixation leads to premature end of trial, where loss of fixation is defined as a recorded eye movement that exits a circular area around the fixation dot of 1.25º radius. To further control for presence of microsaccade artifacts on our data, we performed microsaccade detection on preprocessed and epoched data by an algorithm published by Engbert and Mergenthaler, 2006. In short, a microsaccade is defined as an eye movement that has a velocity of at least six median-based standard deviation, for a duration of at least 12 ms, and an amplitude between 0.1º and 1º. Trials with microsaccades were discarded of further analysis.

1.10 Independent EEG sources by Independent Component Analysis

An Independent Component Analysis (ICA) separates a linear mixture of signals by maximizing statistical independence between signals (Hyvärinen and Oja, 2000; Hyvärinen 2013). In this paper, the statistical independence is measured by the mutual information, so that the neural sources have a maximal mutual information (infomax ICA, Bell and Sejnowski,1995),

implemented within the 'runica' function at the EEGLab toolbox (Delorme and Makeig, 2004). Further analysis on this study are performed on the ICA level.

1.11 Dipole localization on neural sources

Source analysis is based on a physical forward model, which describes electrical relation between sources and EEG electrodes, so that, given neural sources scalp maps and a known geometry of the conductive media (headmodel), we aim to find the best approximation G that describes the relationship between our headmodel m and the observed data D, so that $D = G(m)$. In this paper, dipole localization of neural sources was performed with DIPFIT plugin on EEGLab toolbox (Delorme and Makeig 2004).

In short, we used a standard boundary element model as a headmodel from the Montreal Neurological Institute (BEM-MNI model, Fuchs et al., 2002). The headmodel was derived from an averaged magnetic resonance image and consisted of a total of 4770 nodes or source locations, describing the cortical envelope, the skull and the skin. Using a standard model leads to a source localization as accurate as individual BEM headmodels and more accurate than spherical shell approximations (Fuchs et al., 2002). Locations of the 64 electrodes and 3 fiducial points (naison, left and right earlobe) were measured per subject before EEG recordings (Polaris Vicra, NDI). Those individual electrode locations were aligned to a standard MRI through the location of the fiducials (Fuchs et al., 2002) and mapped to the headmodel by SPM8. For one subject for which the individual electrode locations were not available, BESA headmodel was used.

Source localization in this paper was applied to neural sources obtained by ICA (to its projections on the scalp surface). Accuracy on the localization of the neural sources depends on several factors such as the amount of environmental noise present on the signal, the extend of the neural sources or interference between them. Specifically, source localization error extended from 4.1-20 mm (Acar and Makeig, 2013), so that the quantitative localization of the dipoles provides an approximation for the exact location of the underlying source. Applying source analysis on ICA sources instead of applying it to EEG signals reduces several of the factors that add errors on the dipole localization (e.g. environmental noise, non-linear interference between sources, etc...), increasing accuracy on the source localization (Tarkiainen et al., 2003).

1.12 Detection of task-related neural sources

The goal of this method is to select neural sources that are associated with our stimulus of interest without any prior assumptions on the data (naive Bayes classifier). Generally, our goal is to compute the likelihood $p(D|c_i)$, namely, the probability that a set of data $D = \{x_1^t, x_2^t, \ldots, x_n^t\}$ belongs to a category $C = \{c_1, c_2\}$ where n is the trial number, t the time point at which the likelihood is computed, and C is a binary behavioral condition of interest. For example, if the goal is to find neural sources that associate with the appearance of stimulus, the classification is a binary classification problem $C = \{0,1\}$, where 1 indicates the presence of stimulus. Likewise, other behavioral conditions of interest can be reduced to binary classification problems, such as lateralization of visual stimulation ($C = \{'left', 'right'\}$), or with the direction of eye movement ($C = \{'up, 'down'\}$).

The likelihood for binary categories was computed via logistic regression (Fahrmeir and Tutz, 2001; Bishop, 2007). In short, logistic regression estimates the relationship between predictors variables and the categorical outcomes c_i, mentioned before. The predictor variables of our logistic regression will be the discrete wavelet transform of the neural sources data, so that

$D = \{x_1^t, x_2^t, \ldots, x_n^t\}$ is transformed on the frequency domain such that $\widetilde{D} = \{\tilde{x}_1^t, \tilde{x}_2^t, \ldots, \tilde{x}_n^t\}$. In other words, the logistic regression problem aims to find a set of parameters $\widetilde{w}$ that will establish the following relation: $C = w\,\widetilde{D}$, where C are the categorical outcomes for a time t and $\widetilde{D}$ is the frequency decomposition of a neural source at time t.

To further reduce the dimensionality of our model, we included a L1 or Lasso regularization term on the linear regression (Bishop, 2007). In short, within logistic regression, L1 regularization introduces a penalty term $\alpha|w|$ on the optimization problem, and that results on forcing some of the parameters to have a zero weight. The penalty term is weighted by the hyper-parameter α. To choose the hyper-parameter α, we trained a set of models with α logarithmically spaced between $[0.01, 0.3]$. We choose the hyper-parameter α that lead to the model with highest likelihood (Friedman et al., 2010). Fitting the data for logistic regression and Lasso regularization is performed by glmnet (Friedman et al., 2007).

Evaluation of the classification performance is computed through the Maximum A Posteriori estimate, so that $C_{MAP} = \mathrm{argmax}_C\, p(\widetilde{D}|c_i)$ (Bishop, 2007). Concatenating the MAP at every time step t is what we call the prediction trace (see Figure 2A for an example).The model classification accuracy is validated through repeated random sub-sampling validation, for 100 iterations and a split of the data D into 70% training trials and 30% validation trials, so that for each neural source we estimate 100 prediction traces, which are then averaged as to reduce the bias of the model (Kohavi, 1995). By randomizing categorization labels (random labels were generated through random permutation of the trial number), we obtain the likelihood function of the null-hypothesis, which is, the probability distribution that a dataset $\widetilde{D}$ belongs to a random class c_i (surrogate data, see Figure 2A for an example). To this end, repeated random sub-sampling validation, for 100 iterations and a split of the data D into 70% training trials and 30% validation trials, so that for each surrogate source we estimate 100 prediction traces, from which we obtain a probability distribution of the null hypothesis. The probability distribution of the null-hypothesis was used to compute significance level of the categorization performance, so that if $p(\widetilde{D}|c_i)$ is outside the 99% confidence interval of the distribution of the null-hypothesis, the categorization performance is considered significant (p<0.01; Bakeman and Robinson, 2005).

1.13 Clustering of task-related ICA neural sources and Multiple Comparison Correction

To identify sets of equivalent neural sources across subjects, we cluster them based on their prediction traces. Such clustering was performed by k-means, a distance-based algorithm where n observations are clustered into k groups by minimizing the distance among observations (Bishop, 2007). In this paper, neural sources were clustered for $k = [2,3,\ldots,20]$. The k was chosen to be the smallest k where no qualitative difference on the clusters was observed (visualizing dipole location, ERP and spectrogram). To further correct for multiple comparison, we performed a cluster-based analysis of the prediction traces, based on temporal proximity (Maris and Oostenveld, 2007).

1.14 Spectral Decomposition

We used two different methods to perform the time-frequency decomposition of neural signals. Both time-frequency decompositions were computed on epoched data (-1 to 2 sec relative to event of interest). The number of trials for which the spectral decomposition is computed was kept to 90 trials per condition (contour/non-contour) per subject. Spectral analysis for the spectrograms was computed by the multitaper method for high frequency bands (30-120 Hz), which provides a way to control bias and variance of the spectral estimation by using multiple Slepian tapers (Percival and Walden, 1993), with an starting 400

ms length and a frequency bandwidth of 6 Hz, in steps of 2 Hz. Spectral analysis on frequency bands between 5 and 30 Hz was computed through a continuous wavelet transform using Morlet wavelets, with a width of four cycles per frequency, and steps of 2 Hz. Both estimations were computed by the ft_freqanalysis function on FieldTrip (Oostenveld et al., 2007). Finally, all spectrograms are presented as a power change in respect to the baseline (-0.5 to 0 sec relative to event of interest), and then averaged over trials and subjects. Spectral analysis for the detection of task-related neural sources is performed through discrete wavelet transform (DWT) using Daubechies wavelets of order 4 (Addison, 2002). Discrete wavelet transform with Daubechies wavelets is commonly used within signal processing and economic forecasting community since it is specifically designed for discrete non-stationary time signals (Reis et al., 2005; Ababneh et al., 2013). In short, the discrete wavelet transform scales the original signal s into the sum of individual signals $s_f(t)$, so that $s = \sum_{f=1}^{F} s_f(t)$. The decomposition leads to 5 individual signals $s_f(t)$ that are band limited, with center frequencies that scale as power of 2 (namely, 5.6, 11.16, 22.32, 44.6 and 89.3 Hz mean frequency). The dynamics of the original signal are completely preserved within the decomposed signals, so that summing up a subset of the individual signals $s_f(t)$ reconstructs the dynamics of that restricted frequency range. Instantaneous amplitude and phase of each of the individual signals $s_f(t)$ was computed. The imaginary part of the signal $s_H(t)$ is computed by the Hilbert transform (hilbert.m function of MatLab). The instantaneous amplitude $A_f(t)$ for each individual signal computed as $A_f(t) = \sqrt{\left[s_f(t)\right]^2 + [s_H(t)]^2}$ and the instantaneous phase $\Phi(f,t)$ for a frequency interval f is computed by $\Phi(f,t) = tan^{-1}\left(\frac{s_H(t)}{s_f(t)}\right)$ (angle.m function of MatLab).

1.15 Phase locking analysis

Phase synchronization estimates were computed on the instantaneous phase obtained from the Daubechies spectral decomposition as described in Lachaux et al., 2000, so that the time-resolved Phase Locking Value for a frequency f at time t is defined as:

$$PLV(f,t) \ = \left| \frac{1}{L} \cdot \sum_{k=1}^{L} \exp\,(i\ \Phi_{\mathrm{a,b}}) \right|$$

where L corresponds to trial number and $\Phi_{\mathrm{a,b}}$ is the phase difference between signals a and b at frequency interval f, so that $\Phi_{\mathrm{a,b}}(f,t) = \Phi_{\mathrm{a}}(f,t) - \Phi_{\mathrm{b}}(f,t)$. The phase difference $\Phi_{\mathrm{a,b}}(f,t)$ and the PLV was computed between all pairs of neural sources (so that $a, b \in [1, N]$ for $N = 64$ number of neural sources) for each of the 5 frequency intervals obtained by the DWT. This leads to a total of $N(N-1) \times f$ phase synchronization values. Furthermore, phase difference and PLV were computed for two behavioral conditions (contour and non-contour trials), so that for each condition we have a phase synchronization estimation for each neural source pair $N(N-1)$, at each frequency interval f. As the trial number influences the estimation of phase-differences, the trial number L is kept to 90 trials for each PLV conditions. Those 90 trials were selected randomly from the available set of trials per subject.

1.16 Long-Range synchronization networks - statistics on phase locking analysis

Statistical testing of significance of PLV difference is performed through cluster-based permutation test (Maris and Oostenveld, 2007). In short, our goal is to test the following null hypothesis: phase locking value difference between contour/non-contour trials occurs by chance.

Through Monte Carlo resampling, the distribution of phase locking difference of the null-hypothesis is obtained (phase locking value difference of surrogate data). Note that the

distribution of the surrogate phase locking difference follows a Gaussian distribution and that this procedure will be done to every time point, given that the permutation test considers every time point an independent and identically distributed observation (Maris and Oostenveld, 2007). This implies that we can apply a student's t-test to estimate whether the phase locking value difference between contour and non-contour trials is statistically significantly different from surrogate phase locking differences. In other words, the observed PLV difference is rejected if it is contained within the 99% of the null-hypothesis PLV differences (p<0.01). This procedure results in that each neural source has a set of significant phase-locking values associated to other neural sources, corrected for multiple comparison. The average number of connections per neural source, can be visualized in Supplemental Figure S3; from all possible neural source pairs, the 2.17% of them are synchronized within theta frequency (5.6 Hz), while the 2.47, 3.54, 5.05 and 8.34% synchronize at alpha (11.16 Hz), high beta (22.32 Hz), low gamma (44.6 Hz) and high gamma (89.3 Hz) frequency intervals, respectively. Following, we correct for baseline fluctuations by z-score: we estimate whether the t-statistics are significantly different relative to its baseline by z-score. In short, the z-score determines the number of standard deviation of an observed data x is above the mean, so that $z = \frac{x-\mu}{\sigma}$, where μ corresponds to the population mean and σ corresponds to the standard deviation of the population. Both μ and σ were computed from baseline t-stats (-0.5 to 0 s before event of interest). This procedure results in that each neural has a set of significant phase-locking values associated to other neural sources, corrected for baseline values.

Next, we proceed by analyzing phase-synchronization between particular subsets of neural sources. Specifically, we performed two different analysis: we first analyze synchronization pattern of parietal neural sources (PCO) to frontal neural sources (FCO), and second, we analyze the synchronization pattern between parietal (PCO) and frontal sources that are not FCO (namely, the frontal neural sources that are not selective of contour/non-contour, the FNC cluster, see section 1.17 below). The procedure to statistically analyze the phase locking synchronization pattern between a subset of neural sources can be visualized in Supplemental Figure S4, and the result of this analysis is presented in Figure 4 on the main text. The Figure 4 represent the accumulated t-statistics of all the significant phase-locking differences between contour and non-contour trials, colored in the background. In other words, the cumulative sum (cumsum.m from MatLab) t-statistics can be only be positive if the t-statistics of all the sources considered is also positive. To increase clarity, these plots also present the average t-statistics over the considered neural sources (black line), corrected for baseline values. Note that through the analysis of phase locking of a subset of neural sources, we are indeed performing a clustering-like procedure as described in Maris and Oostenveld, 2007. In other words, by analyzing the phase synchronization between parietal-frontal areas (Figure 4) the t-statistics for a certain time point t has a significance p-value of $p^{(32)}$.

1.17 Selection of frontal neural sources FNC

Long-range phase-synchronization was analyzed between parietal frontal sources PCO and frontal sources that are not FCO, the cluster labeled FNC. The neural sources that belong to the cluster FNC were selected, in a subject-specific manner, as follows: the frontal lobe was approximated as the cortical area that spans 1/3 of the total brain volume on both the z and y axes, and all the neural sources within this volume were kept as frontal neural sources. In particular, if z_{max} is the z coordinate maximal volume, and y_{max} the y coordinate brain volume, the neural sources within frontal cortex are those which lie within the range of [z_{max}, $z_{max} - {}^{1}/_{3} \times z_{max}$] and [$y_{max}$, $y_{max} - {}^{1}/_{3} \times y_{max}$] coordinates. From the set of the neural sources that lie within this volume, those that have been previously selected as FCO were

extracted. The resulting neural sources were used for further analysis as the cluster FNC, see Figure S5 to visualize the resulting neural sources, for all subjects. The validity of our approximation method was estimated by obtaining the anatomical location of the centroid of the neural sources for each subject and checking whether it lies within the frontal cortex, given its Talariach coordinates, see Table S1. For all subjects, the approximated FNC neural sources lie within the frontal cortex, either at Broadmann area 10 or Broadmann area 9. When considering the neural sources of all subjects, what we consider the cluster FNC, the centroid of the resulting FNC lies within the frontal cortex.

2. Supplementary Figures

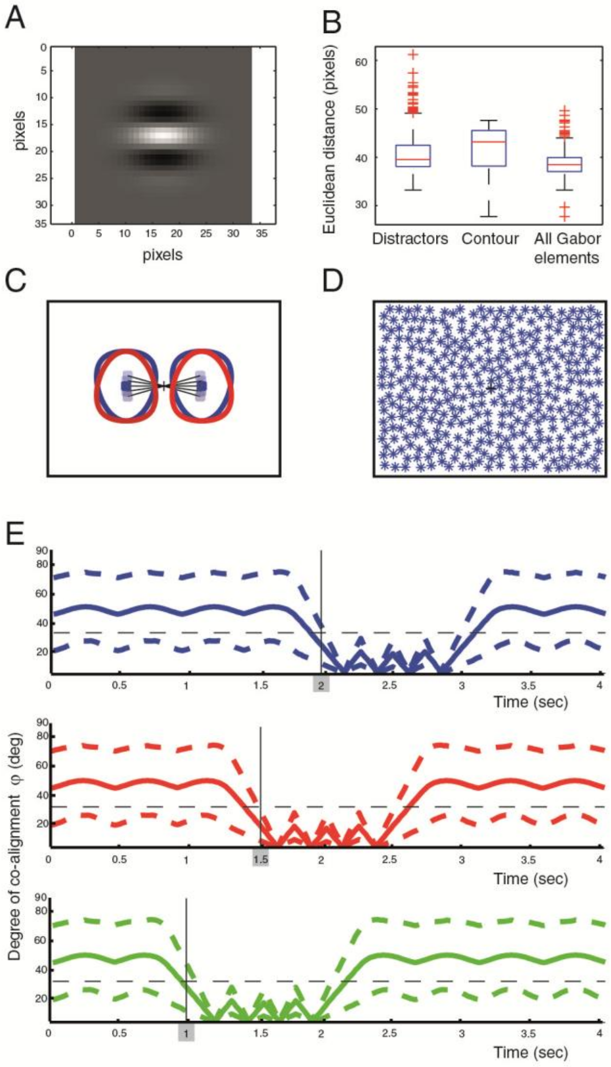

Figure S1: Stimulus for the behavioral task. *A) Gabor element, with an envelope of 3.5cycles, spanning 17 pixels (0.5 degree of visual angle). B) Distance between nearest Gabor elements, comparing the set of background Gabor elements, Gabor elements forming the contour, and all Gabor elements of a stimulus frame. C) Spatial locations where a contour can appear on a single trial, color coded to increase clarity. Subjects had to report the pointing direction of the contour: up/down. Contours appear left/right of the hemifield (50% each) on 5 different locations from horizontal line, marked as blue shadow (at either -16, -8, 0, 8, 16 degrees from horizontal line, where 0 is the horizontal line. Note that 8 deg in visual field corresponds to at least 24 pixels distance) D) Location of Gabor elements on a stimulus frame, where each Gabor element position is marked with a star. The stimulus shown contains a contour. E)Temporal evolution of the degree of co-alignment φ of Gabor elements involved contour for three different trials (see text for details). Appearance of contour or contour onset is marked with black vertical line, while the gray shadow on the timeline presents the range of contour visibility (± 66 ms around contour onset).*

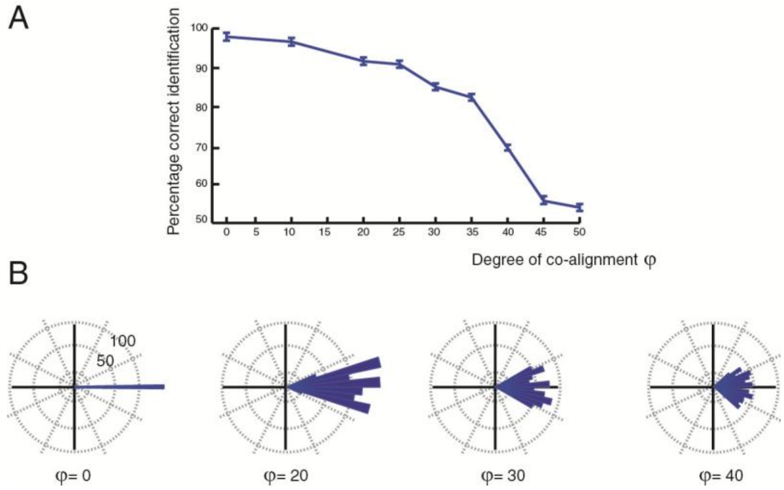

Figure S2: Psychometric curve of contour identification for morphing stimulus *A) Performance on contour identification as a function of the degree of contour co-alignment . B) Distribution of angle distance in respect to figure path for different degrees of co-alignment φ.*

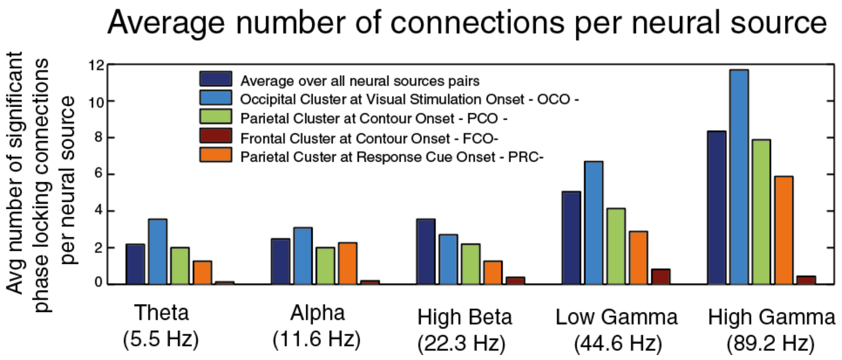

Figure S3. Average number of connections per neural source*, at different frequency intervals f, for different subsets of neural sources (see text for details). The occipital neural sources associated to visual stimulation are presented in light blue. In green, the parietal neural sources associated to contour integration, while dark red marks the frontal neural sources associated to contour integration. Finally, in orange, the parietal neural sources associated to saccade planning (see section Local synchronization control tests in the main manuscript).*

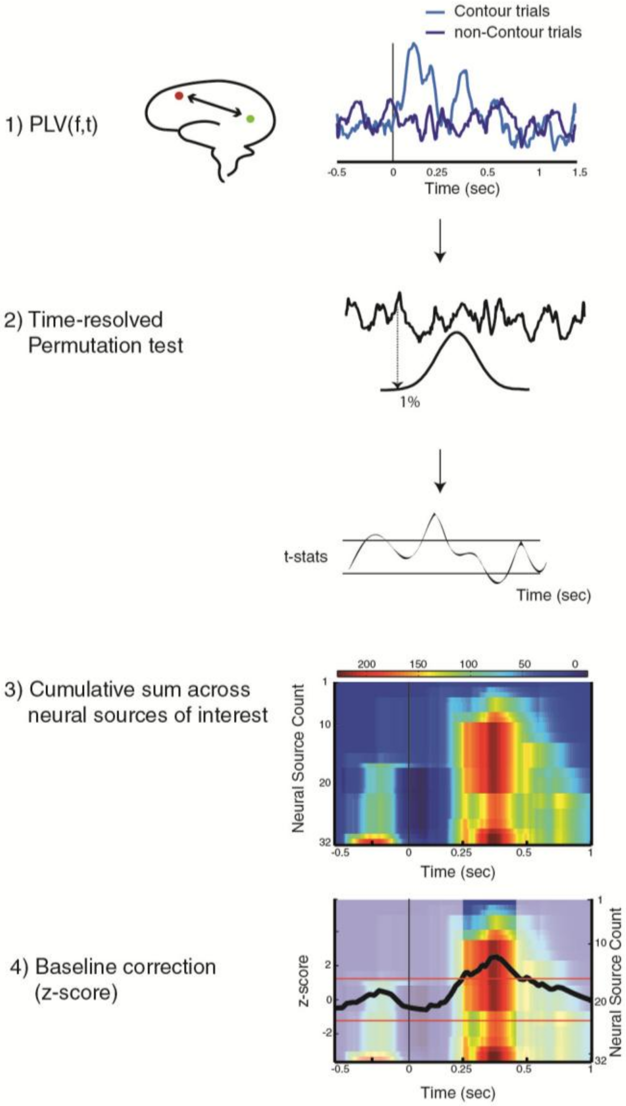

***Figure S4. Statistical analysis of phase-locking networks**, divided in 4 steps. 1) Computation of phase locking value for each source pair, at each frequency interval f, for two behavioral conditions: contour and non-contour trials. 2) Compute statistical significance of time resolved phase-locking difference by a permutation test (see text for details). 3) Select subset of neural sources of interest (e.g. spatial location or behavioral association) and accumulate t-statistic of significant phase locking values to other sources, across subjects. Finally, baseline correction with z-score in 4).*

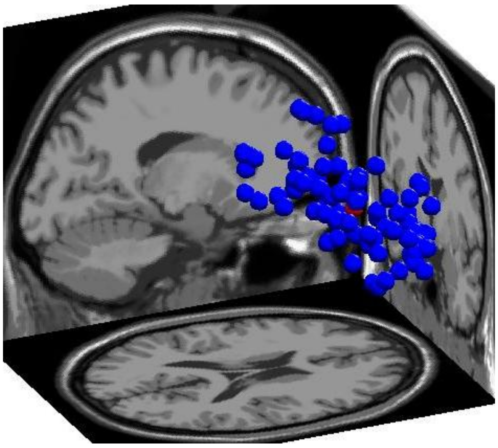

Figure S5: Dipole localization of frontal neural sources non-associated to contour integration (all frontal except those involved in cluster FCO) over all subjects

Table S1: Coordinates in Talariach space (Lancaster et al., 2000) of frontal neural sources non-associated to contour integration (all frontal excpet those involved in cluster FCO), and their localization in the brain. Averaged over all subject and in a subject-specific manner

Cluster Name	x, y, z Talariach coordinates	Anatomical Structure	Broadmann area
**All Subjects**	**(-1.47, 47.84, 11.39)**	**Frontal Lobe<br>Medial Frontal Gyrus**	**Broadmann area 10**
**Subject 1**	(-12.87, 43.63, 13.71)	Frontal Lobe<br>Medial Frontal Gyrus	Broadmann area 10
**Subject 2**	(14.76, 47,14, 10.77)	Frontal Lobe<br>Medial Frontal Gyrus	Broadmann area 10
**Subject 3**	(-23.99, 28.48, 27.98)	Frontal Lobe<br>Medial Frontal Gyrus	Broadmann area 9
**Subject 4**	(-21.97, 39.19, 13.27)	Frontal Lobe<br>Medial Frontal Gyrus	Broadmann area 10
**Subject 5**	(5.12, 48.97, 9.47)	Frontal Lobe<br>Medial Frontal Gyrus	Broadmann area 10
**Subject 6**	(-22.29, 44.15, 28.12)	Frontal Lobe<br>Medial Frontal Gyrus	Broadmann area 9
**Subject 7**	(8.40, 39.00, 6.99)	Frontal Lobe<br>Medial Frontal Gyrus	Broadmann area 10
**Subject 8**	(9.03, 37.24, 6.12)	Frontal Lobe<br>Medial Frontal Gyrus	Broadmann area 10
**Subject 9**	(4.36, 47.53, 3.41)	Frontal Lobe<br>Medial Frontal Gyrus	Broadmann area 10
**Subject 10**	(10.81, 56,23, 10.85)	Frontal Lobe<br>Medial Frontal Gyrus	Broadmann area 10
**Subject 11**	(3.66, 40.50, 28.52)	Frontal Lobe<br>Medial Frontal Gyrus	Broadmann area 9
**Subject 12**	(-11.70, 36.90, 11.84)	Frontal Lobe<br>Medial Frontal Gyrus	Broadmann area 10
**Subject 13**	(-37.08, 39.00, 12.60)	Frontal Lobe<br>Medial Frontal Gyrus	Broadmann area 10
**Subject 14**	(-0.57, 62.62, 8.20)	Frontal Lobe<br>Medial Frontal Gyrus	Broadmann area 10
**Subject 15**	(14.84, 76.11, 1.50)	Frontal Lobe<br>Medial Frontal Gyrus	Broadmann area 10

3. References

Ababneh, F., Wadi, S. Al, and Ismail, M.T. (2013). Haar and Daubechies Wavelet Methods in Modeling Banking Sector. Int. Math. Forum *8*, 551–566.

Acar, Z.A., and Makeig, S. (2013). Effects of forward model errors on EEG source localization. Brain Topogr. *26*, 378–396.

Addison, N. (2002). The illustrated wavelet transform handbook. Introductory theory and applications in science, engineering, medicine, and finance (Institute of Physics Publishing Bristol and Philadelphia).

Bakeman, R., and Robinson, B.F. (2005). Understanding statistics in the behavioral sciences (Cengage Learning).

Bell, A.J., and Sejnowski, T.J. (1995). An Information-Maximization Approach to Blind Separation and Blind Deconvolution. Neural Comput. *7*, 1129–1159.

Bishop, C.M. (2007). Pattern Recognition and Machine Learning (Springer).

Delorme, A., and Makeig, S. (2004). EEGLAB: an open source toolbox for analysis of single-trial EEG dynamics including independent component analysis. J. Neurosci. Methods *134*, 9–21.

Delorme, A., Sejnowski, T., and Makeig, S. (2007). Enhanced detection of artifacts in EEG data using higher-order statistics and independent component analysis. Neuroimage *34*, 1443–1449.

Engbert, R., and Mergenthaler, K. (2006). Microsaccades are triggered by low retinal image slip. PNAS *103*, 7192–7197.

Fahrmeir, L., and Tutz, G. (2001). Multivariate Statistical Modelling Based on Generalized Linear Models (Springer; 2nd edition).

Friedman, J., Hastie, T., Höfling, H., and Tibshirani, R. (2007). Pathwise coordinate optimization. Ann. Appl. Stat. *1*, 302–332.

Friedman, J., Hastie, T., and Tibshirani, R. (2010). Regularization Paths for Generalized Linear Models via Coordinate Descent. J. Stat. Softw. *33*, 1–22.

Fuchs, M., Kastner, J., Wagner, M., Hawes, S., and Ebersole, J.S. (2002). A standardized boundary element method volume conductor model. Clin. Neurophysiol. *113*, 702–712.

Hess, R.F., Beaudot, W.H., and Mullen, K.T. (2001). Dynamics of contour integration. Vision Res. *41*, 1023–1037.

Hyvärinen, A, and Oja, E. (2000). Independent component analysis: algorithms and applications. Neural Netw. *13*, 411–430.

Hyvärinen, A. (2013). Independent component analysis: recent advances. Philos. Trans. R. Soc. A *371*.

Kohavi, R. (1995). A Study of Cross-Validation and Bootstrap for Accuracy Estimation and Model Selection. Int. Jt. Conf. Artif. Intell. IJCAI.

Lachaux, J.P., Rodriguez, E., Martinerie, J., and Varela, F.J. (1999). Measuring phase synchrony in brain signals. Hum. Brain Mapp. *8*, 194–208.

Mathes, B., Trenner, D., and Fahle, M. (2006). The electrophysiological correlate of contour integration is modulated by task demands. Brain Res. *1114*, 98–112.

Maris, E., and Oostenveld, R. (2007). Nonparametric statistical testing of EEG- and MEG-data. J. Neurosci. Methods *164*, 177–190

Percival, D.B., and Walden, A.T. (1993). Spectral analysis for physical applications (Cambridge University Press).

Plöchl, M., Ossandón, J.P., and König, P. (2012). Combining EEG and eye tracking: identification, characterization, and correction of eye movement artifacts in electroencephalographic data. Front. Hum. Neurosci. *6*, 278.

Reis, A.J.R., Alves, A.P., and Member, S. (2005). Feature Extraction via Multiresolution Analysis for Short-Term Load Forecasting. IEEE Trans. Power Syst. *20*, 189–198.

Oostenveld, R., Fries, P., Maris, E., and Schoffelen, J.-M. (2011). FieldTrip: Open source software for advanced analysis of MEG, EEG, and invasive electrophysiological data. Comput. Intell. Neurosci. *2011*, 156869.

Tarkiainen, A, Liljeström, M., Seppä, M., and Salmelin, R. (2003). The 3D topography of MEG source localization accuracy: effects of conductor model and noise. Clin. Neurophysiol. *114*, 1977–1992.

Yuval-Greenberg, S., Tomer, O., Keren, A.S., Nelken, I., and Deouell, L.Y. (2008). Transient induced gamma-band response in EEG as a manifestation of miniature saccades. Neuron *58*, 429–441.
